# The semantic differential questionnaire format warrants consideration for use in healthcare settings

**DOI:** 10.1007/s11136-026-04198-9

**Published:** 2026-03-08

**Authors:** Ariel Pons, Kate Sneddon, Gillian Whalley, Crispin Jenkinson, David Morley, Sean Coffey

**Affiliations:** 1 Department of Medicine, Otago Medical School, Dunedin, New Zealand; 2 HeartOtago, Department of Medicine, Dunedin School of Medicine, PO Box 56, Dunedin, 9054 New Zealand; 3 Harris Manchester College, University of Oxford, Oxford, UK; 4 Nuffield Department of Population Health, University of Oxford, Oxford, UK

**Keywords:** Semantic differential, Likert, Questionnaire generation, Health-related quality of life

## Abstract

**Background:**

Semantic differential scales, in which respondents rate their relative agreement between two opposing statements, are rarely used in assessment of health related quality of life (QOL). Here we present a use-case study describing our experiences constructing such a scale, part of a larger project to generate a questionnaire, the VALVQ, to measure QOL in heart valve disease (HVD).

**Methods and results:**

Individuals with HVD were identified from echocardiographic databases. Initial questionnaire content was generated from semi-structured interviews with 34 individuals with HVD as well as three family members and five clinical experts. Four methods were used to generate semantic differential items. Firstly, two participants could give opposing points, allowing an item to be generated directly. Secondly, a participant could report a single experience at one extreme, with the research team generating the opposing statement. Thirdly, a participant could report an experience in the middle of two extremes, with two opposing statement generated by the research team. Finally, subtly different items were generated where an obvious corresponding item was not clear, allowing for future psychometric testing to examine item robustness. 64 semantic differential items were generated for pilot testing. 46 valid completed questionnaires were returned. Multiple items required changes due to phrasing of the item content, but no difficulties were reported with the semantic differential format.

**Conclusion:**

The semantic differential scale can be considered for health related QOL research. Further head-to-head comparisons with the more common Likert format will allow assessment of differences in respondent biases between these questionnaire formats.

**Supplementary Information:**

The online version contains supplementary material available at 10.1007/s11136-026-04198-9.

## Abstract (plain language summary)

In medical research, measuring people’s quality of life is important to guide treatment. However, measuring an abstract concept like quality of life is difficult, and using a questionnaire that is not designed suitably could cause researchers to come to wrong conclusions. There’s a format for questionnaires called the ‘semantic differential’: instead of having one statement that people rate their agreement with, it has two opposing statements that people rate their agreement between. We think this format could be beneficial for improving questionnaire quality when measuring quality of life. However, since this format isn’t commonly used, there isn’t much guidance on how to make a questionnaire using one. We developed a semantic differential questionnaire to measure quality of life in patients with heart valve disease, and we found it worked well; we were able to make one without problems, and the people who tested the questionnaire had no problems with the format. We share our process of making our questionnaire, so that other researchers can have a starting point for developing semantic differential questionnaires of their own.

**Supplementary Information:**

The online version contains supplementary material available at 10.1007/s11136-026-04198-9.

## Introduction

All questionnaires are subject to a range of biases. In health research, patient reported outcome measure (PROM) scores are often central to selection of interventions, and therefore it is crucial that any bias be as limited as possible. Examples of biases include:

- Framing bias: The positive or negative emotional framing of a statement affects the respondent’s chance of responding in a positive or negative way
- Acquiescence bias (also known as satisficing): If faced with a burdensome mental task, e.g. a long or complicated questionnaire format, respondents become less likely to answer according to how they actually feel, and more likely to pick a convenient answer [1].
- Social desirability bias: The respondent answers according to how they anticipate others want them to feel, rather than how they feel [2].

There are many more biases [3], but framing and acquiesce bias are of particular relevance when deciding on questionnaire format, since one possible source of bias is the scale used to assess responses. In the most commonly used format, the Likert scale, a single phrase is posed and respondents rate their agreement with this statement, or the frequency with which it applies [4].

**Fig. 1:**
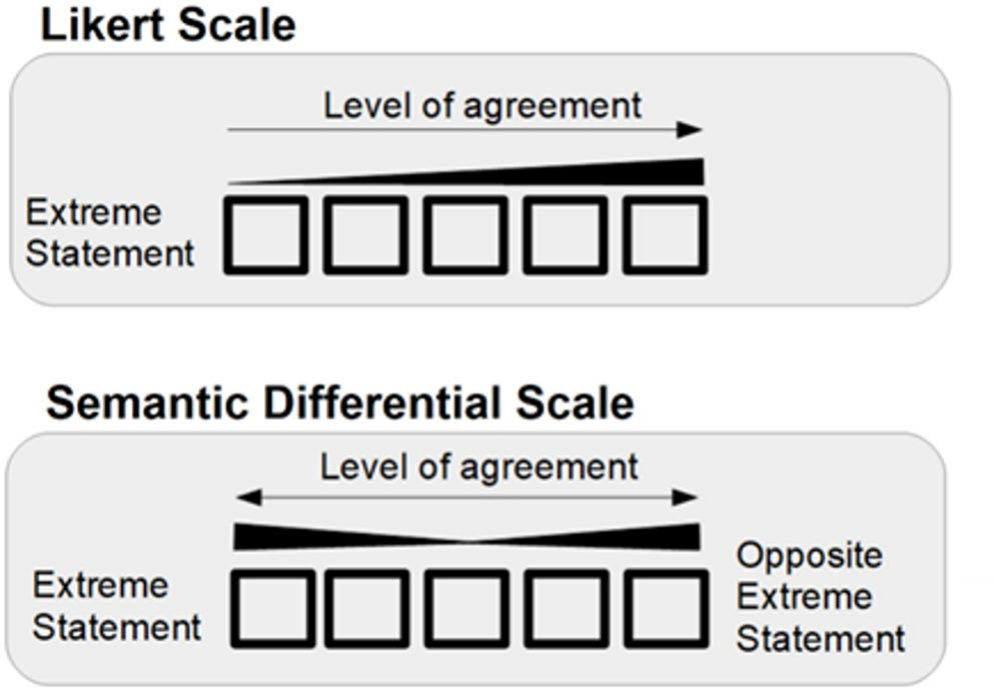
Likert and semantic differential scales

A semantic differential scale is an alternative format. This format has two opposing polar statements, one of which is posed on the left, the other on the right, with a range of positions between them [5]. Respondents note their relative position of agreement between the two statements. The two scales are represented in Fig. 1.

Semantic differential scales are rarely used or considered in health-related quality of life (QOL) PROMs, possibly due to lack of familiarity with the use of the format and its psychometric analysis and interpretation. However, this format has a number of attractive qualities. The Likert scale is likely to be susceptible to framing bias [6], which is a form of bias of particular importance when measuring emotive concepts such as QOL. This framing bias can potentially be reduced when using a Likert format by transforming alternate items into the negative form (e.g. ‘I am happy’ becomes ‘I am sad’), but including both positive and negative items in the same questionnaire is likely to induce respondent error [7]. The semantic differential format, with its positive and negative statements within each item, may reduce framing bias without increasing respondent error.

It is also difficult to be certain what a respondent of a Likert truly means with their response selection: does disagreement with a statement indicate negative association with the statement, or positive association with a separate concept [8]? In contrast, a semantic differential forces respondents to rate their agreement between two specified extremes.

However, a possible criticism of the semantic differential format is that it could be expected to increase response burden. Responding, presumably, requires at least twice the effort of a Likert, since there are two statements to read, and the respondent must perform a more complex mental calculation to determine their relative position between them. This increased respondent burden could be assumed to increase acquiescence bias—though, in practice, the semantic differential format may display less acquiescence bias than the Likert [7]. What is more certain is that generation of a semantic differential increases burden on the researcher team, who have to generate twice the number of statements per item, and additionally ensure they have true bipolarity [9]. Nevertheless, the Likert and semantic differential formats often behave comparably [10], though psychometric procedures for Likerts are much more well-established [11]. Selecting a format is therefore a consideration that must be determined by a research team according to their specific aims and needs. However, due to its infrequent use in the health sciences, clinical researchers often have little exposure to semantic differential formats.

In this article, we describe one part of a questionnaire development process: the conversion of information gathered from interviews to items in a semantic differential scale. We also describe how viable development of a semantic differential scale is in the setting of a health-related QOL instrument.

## Methods

This paper is part of a multiphase project aiming to generate a self-reported questionnaire measuring QOL in HVD, the VALVQ [12]. This study is reported in accordance with the COREQ checklist [13], which is available in the Appendix (Appendix Table 1).

### Study background

The study protocol is described in more detail elsewhere [12], but in summary, the VALVQ aims to measure quality of life (QOL) across the lifespan of people with HVD, both before and after any valve procedures. Heart valve disease is a group of conditions in which one or more of the four valves within the heart either become too leaky (valvular regurgitation) or restrict blood flow (valvular stenosis). The forms of HVD that cause the most morbidity and mortality worldwide are rheumatic heart disease (RHD, which predominantly affects the mitral valve), aortic stenosis (AS), and mitral regurgitation (MR) [14]. Additionally, an increasingly important form of HVD is seen in people with previous valve repair/replacement (VRR). In the initial phases of VALVQ development, we focused on these four conditions.

The first phase of this project consisted of developing semantic differential items through interviews with patients, family members, and clinical experts. The second phase saw this initial questionnaire pilot tested for acceptability to participants, a separate cohort of patients with HVD. This article focuses on the results of these stages. The planned phase three, which details selecting final items and validation testing of the final VALVQ, is described further elsewhere [12].

### Participant identification and recruitment

People with HVD or VRR were identified using the echocardiographic imaging databases in Southern District Health Board and Canterbury District Health Boards, New Zealand, between October 1 to December 31 2019, according to diagnoses on echocardiographic reports. Eligibility was assessed using the electronic patient record to access age, diagnoses lists, and clinic letters discussing their diagnosis. Eligible patients were approached in consecutive order for potential recruitment.

### Inclusion and exclusion criteria

To be included in this project, individuals with HVD had to have significant, primary heart valve disease or a repair/replacement of a damaged valve as their primary diagnosis, as well as awareness of their diagnosis. For the first and second phases, individuals were excluded if they were younger than 18, had a terminal illness or cognitive/mental impairment, had a severe comorbidity or comorbidity significant enough to be expected to have a greater effect on the person’s QOL than their HVD, or were pregnant (due to the changes in heart and valvular function during pregnancy). Family members of recruited patients in phase one had to be over the age of 18 and have awareness of the patient’s HVD.

Additionally, individuals were excluded from phase one if they had a clinically significant cardiac comorbidity other than well-controlled atrial fibrillation, in order to allow clearer identification of the relationship between QOL and HVD. However, since phase two only required questionnaire functionality to be tested, phase two did not have this additional exclusion criterion.

Family members of HVD participants were identified by the participant in their interviews, and clinical experts were identified by the primary investigator (SC).

### Data collection

In phase one, participant interviews were conducted online using video calling platforms (Skype or Zoom) or a phone call, as per the participant’s preferences, due to nationwide COVID-19 lockdowns occurring at the time. Interviews were semi-structured and collected individual’s experience of their HVD and QOL in detail, and continued until saturation was reached. The interview questions asked are available in the Appendix (Appendix Table 2). Initial questions were generated from a prior literature review [15]. Questions were removed if multiple participants found questions irrelevant. Questions were added if multiple participants spontaneously volunteered discussion on the topic.

Interviews were conducted by AP, then a medical and PhD student, after completing an online course on focus groups and qualitative interviewing. Participants were sent an information sheet and consent form before participation, and they were aware their results would be used for both papers and AP’s PhD thesis. Audio recording was used to generate transcripts from interviews.

In phase two, participants were contacted by phone and post, and were posted a paper copy of the questionnaire as well as a link to complete an online version on Redcap [16, 17]. Each item had space for respondents to record comments on the item in addition to responding to the item itself. At the end of the questionnaire, respondents were asked what they liked about the questionnaire, what they disliked or found confusing, and anything they thought that was important that was not included in the questionnaire. The complete questionnaire, along with respondent feedback in the pilot testing phase, is available in the Appendix (Appendix Table 3). We aimed for a minimum of 20 respondents for pilot-testing [12].

### Analysis

The methodological association underpinning this project was phenomenology [18], with the patients’ lived experience of HVD and the resulting impacts on their lives being the phenomenon of interest.

The first phase was entirely qualitative and aimed to capture all aspects of QOL in HVD and so contained all data that had been gathered from the interviews, even if the resulting items were potentially irrelevant or redundant. Descriptive data was collated from the questionnaires, and each perspective/experience reported by respondents was identified as a topic. Each topic was formatted into an item format (e.g. a respondent reporting they felt tired was re-formatted into the item, ‘I feel tired’).

Since interviews continued until saturation was reached, many respondents reported the same topics, and so these were combined. However, many respondents reported slightly different aspects of the same or a very similar topic. In these cases, each aspect was retained and added as a questionnaire item (e.g. the questionnaire contains the items, “I have a positive attitude”, “I feel motivated”, and “I can achieve big things”). If respondents reported exactly opposing experiences to each other, e.g. “walking is easy” and “walking is difficult”, these were combined into one single questionnaire item using the semantic differential format.

This resulted in an item list of 255 items, generating the first initial questionnaire. As this would make an impractically long questionnaire for initial testing, multiple rounds of face validity were required to reduce this to a manageable 64 items in an initial questionnaire for pilot testing. This analysis was done by the primary author, then a medical student (AP), a clinical cardiologist (SC), a clinical cardiac sonographer (GW), and a data analyst (KS).

In the second phase, pilot testing, the methodology was primarily descriptive, with mixed qualitative and quantitative assessments. Overall analyses reported participant feedback on the overall questionnaire, proportion of respondents able to answer all questions, and number of items left blank per item and per respondent. Individual item analyses were the distribution of respondent’s answers, respondent feedback, and changes made as a result for each item. All quantitative analysis (i.e. participant demographics) were done in R [19].

Generating the final questionnaire, by rigorous testing for item selection and removal, and validation assessments, are outside the scope of this paper and are to be analysed in the third phase of the project.

## Results

### Participant demographics

In the first phase (interviews), 125 people were identified as eligible and 34 (27%) participated. Of non-participants, only 20% actively declined participation; 80% did not respond to attempted contact. There was no statistically significant difference in the age, gender, HVD type, HVD severity, or ethnicity of participants and non-participants (t-test p < 0.05). Of the HVD participants, 7 had AS, 7 had RHD, 9 had MR, and 11 had VRR. Five clinical experts and three family members of HVD participants were interviewed. The clinical experts, recruited from New Zealand (NZ) and Australia, consisted of a clinical cardiologist, an imaging cardiologist, two structural interventional cardiologists, and a general practitioner specialising in the care of rural patients with RHD (i.e. a total of 42 interviews).

Of the 34 HVD participants, the median age was 67 (min 19, max 93) years and 19 (56%) were female. The ethnicity of 28 (82%) of the population studied were NZ European (Table 1: Phase one participant demographics).

**Table 1:** Phase one participant demographics

	Total sample	AS	MR	RHD	VRR
Number	34	7	9	7	11
Median age (range)	67 (19–90)	62 (55–78)	70 (58–82)	47 (19–83)	73 (60–60)
Male sex (%)	15 (44.1)	6 (85.7)	3 (33.3)	1 (14.3)	5 (45.5)
Ethnicity (%)					
NZ European	28 (82.4)	6 (85.7)	8 (88.9)	3 (42.9)	11 (100)
Samoan	2 (5.9)	0	0	2 (28.6)	0
Māori	1 (2.9)	0	1 (11.1)	0	0
European	1 (2.9)	1 (14.3)	0	0	0
Fijian	1 (2.9)	0	0	1 (14.3)	0
Filipino	1 (2.9)	0 0	0	1 (14.3)	0
HVD severity (%)					
Mild	2 (5.9)	0	0	2 (28.6)	NA
Moderate	6 (17.6)	4 (57.1)	1 (11.1)	1 (14.3)	NA
Moderate-severe	6 (17.6)	1 (14.3)	3 (33.3)	2 (28.6)	NA
Severe	7 (20.6)	2 (28.6)	5 (55.6)	0	NA
Comorbidities (%)					
Ischaemic heart disease	28 (60.9)	2 (33.3)	11 (61.1)	4 (66.7)	11 (64.7)
Atrial fibrillation	19 (41.3)	4 (66.7)	8 (44.4)	2 (33.3)	5 (29.4)
Heart failure	6 (13.0)	1 (16.7)	2 (11.1)	0 (0.0)	2 (11.8)
COPD/asthma	6 (13.0)	1 (16.7)	1 (5.6)	2 (33.3)	1 (5.9)
Prior TIA/fully-recovered stroke	3 (6.5)	1 (16.7)	2 (11.1)	0	0 (0.0)
Prior cancer	3 (6.5)	0	1 (5.6)	1 (16.7)	1 (5.9)
Diabetes	9 (19.6)	0	4 (22.2)	0 (0.0)	4 (23.5)
Joint pain/replacement	7 (15.2)	0	4 (22.2)	1 (16.7)	3 (17.6)
Anxiety/depression	4 (8.7)	0	1 (5.6)	0	3 (17.6)
Chronic kidney disease	1 (2.2)	0	0 (0.0)	0	1 (5.9)

AS, Aortic stenosis; COPD, Chronic obstructive pulmonary disease; HVD, Heart valve disease; MR, Mitral regurgitation; NZ, New Zealand RHD, Rheumatic heart disease; SD, Standard deviation; TIA, Transient ischaemic attack; VRR, Valve replacement/repair

In the second phase (pilot testing), 111 potential participants were identified and were posted questionnaires. Fifty questionnaires were returned though four people did not return consent forms, leaving 46 for analysis and follow-up (see Table 2: Phase two participant demographics). Most participants either had MR (39%) or VRR (37%). Median age was 77 years, 54% were male, and 89% were NZ European. Most HVD participant had severe rather than mild forms of heart valve disease, save for RHD participants, who had more cases of mild native valve disease. 61% of all participants had ischaemic heart disease/coronary artery disease as comorbidities and 41% had atrial fibrillation/flutter.

**Table 2:** Phase two participant demographics

	Total sample	AS	MR	RHD	VRR
Number	46	6	18	6	17
Mean age (SD)	76.8 (9.1)	82.6 (5.0)	74.4 (11.2)	78.0 (8.1)	75.7 (7.6)
Male (%)	25 (54.3)	4 (66.7)	10 (55.6)	3 (50.0)	9 (52.9)
*Ethnicity (%)*					
NZ European	41 (89.1)	6 (100)	17 (94.4)	5 (3.3)	14 (82.4)
European	2 (4.3)	0	1 (5.6)	0	1 (5.9)
Other	3 (6.5)	0	0	1 (16.7)	2 (11.8)
*Severity (%)*					
Mild	1 (2.2)	0	0	1 (16.7)	
Mild-moderate	2 (4.4)	0	2 (11.1)	0	
Moderate	6 (13.3)	1 (16.7)	4 (22.2)	1 (16.7)	
Moderate-severe	8 (17.8)	3 (50.0)	5 (27.8)	1 (16.7)	
Severe	10 (22.2)	2 (33.3)	7 (38.9)	1 (16.7)	
*Comorbidities (%)*					
IHD/CAD	28 (60.9)	2 (33.3)	11 (61.1)	4 (66.7)	11 (64.7)
Atrial fibrillation/flutter	19 (41.3)	4 (66.7)	8 (44.4)	2 (33.3)	5 (29.4)
Diabetes	9 (19.6)	0	4 (22.2)	0	4 (23.5)
Joint pain/replacement	7 (15.2)	0	4 (22.2)	1 (16.7)	3 (17.6)
Heart failure	6 (13.0)	1 (16.7)	2 (11.1)	0	2 (11.8)
COPD/asthma	6 (13.0)	1 (16.7)	1 (5.6)	2 (33.3)	1 (5.9)
Prior TIA/fully-recovered stroke	3 (6.5)	1 (16.7)	2 (11.1)	0	0
Cancer	3 (6.5)	0	1 (5.6)	1 (16.7)	1 (5.9)
Anxiety/depression	4 (8.7)	0	1 (5.6)	0	3 (17.6)
Chronic kidney disease	1 (2.2)	0	0	0	1 (5.9)

AS, Aortic stenosis; CAD, Coronary artery disease; COPD, Chronic obstructive pulmonary disease; HVD, Heart valve disease; IHD, Ischaemic heart disease; MR, Mitral regurgitation; NZ, New Zealand RHD, Rheumatic heart disease; SD, Standard deviation; TIA, Transient ischaemic attack; VRR, Valve replacement/repair

Sixteen potential participants did not return completed questionnaires but did provide feedback when contacted by phone. The median age was 75, 50% were male, those with HVD mostly had severe valve disease, and all were of NZ European ethnicity.

Participants (both those who returned questionnaires and those who only provided feedback over the phone) were older than non-participants (76 versus 68, t-test p = 0.002), but did not significantly differ in HVD group, sex, or ethnicity.

### Questionnaire generation

Interviews were conducted between the 15th of March 2020 and the 2nd of October 2020 and took between 10 and 35 min. Interviews with clinicians were approximately 30 min long. Interview transcripts were used to generate a list of 374 features related to QOL. When transformed into questionnaire items in a semantic differential format, some features could be paired as opposing statements and a list of 255 items was generated. The following themes were identified: physical limitation, especially limitations relative to a prior 'normal'; symptom burden, especially fatigue; sleep, social stressors/support, medication burden, treatment concerns, individuals' perception of their disease and identity, emotional status, and interactions with the healthcare system. The research team met to discuss and remove items considered to be either not indicative of QOL or likely insensitive to changes in HVD status, leaving a 64-item questionnaire which was then pilot tested over October to November 2020. Items and the interview findings they were developed from are available in the Appendix (Appendix Table 4).

The use of a semantic differential allowed for a wider range of methods in which features could be transformed into items, rather than a Likert where respondents’ experiences and perspectives can only be summated into a single statement. These methods are shown in Table 3: Semantic differential item generation and described using participant quotes directly where able, or a summation of participant’s quotes when required for clarity or brevity.

**Table 3:** Semantic differential item generation

Participants’ experiences	Negative statement		Positive statement
*Item generated from opposing points*			
Constant pain, even at rest No pain	I'm in pain, even when I'm sitting and resting	□□□□□	I'm not in pain
Walking long distances easily Walking even short distances is very difficult	Walking is difficult	□□□□□	Walking is easy
Needing to take things slowly but having time to rest Rushed, overwhelmed	I feel overwhelmed at work	□□□□□	I can take time for a rest at work if I need it
*Item generated from one extreme point*			
Would be very embarrassing to be dependent on others	I feel embarrassed because I need help even in basic activities	□□□□□	I have no need to feel embarrassed about my health
Enjoying challenging mental stimulation	Everything is boring	□□□□□	I have mental stimulation and challenges
Good relationships with colleagues making things easier	My work colleagues make things harder	□□□□□	I have good relationships with my work colleagues
*Item generated by forming extremes around a middle point*			
Sometimes having trouble keeping up with friends on group trips	I am unable to keep up with my friends when we do things together	□□□□□	I am able to keep up with my friends when we do things together
Limited by HVD and unsure how long the limitations will be present	I expect my life and activities to be limited by my disease for a long time	□□□□□	I am not limited by my disease, or I expect my limitations to be over soon
Getting to and from hospital can be difficult	Getting to hospital when I need it is a lot of trouble	□□□□□	Getting to hospital when I need it is easy
*Multiple similar items generated from a range of experiences*			
QOL = “doing what you used to” QOL = “doing your own thing” QOL = “able to do the things I like”	I’m unable to do my usual activities	□□□□□	I’m able to do my usual activities
	I’m unable to do the things I like doing	□□□□□	I’m able to do the things I like doing
	I’m not able to do my own thing	□□□□□	I’m able to do my own thing
Missing friends (COVID-19 lockdown) Appreciates being amongst people Talking to others helps QOL When in hospital, camaraderie helps; it’s “extra tough” with no visitors	I miss my friends	□□□□□	I'm able to spend as much time as I want with my friends
	I feel isolated and unsupported	□□□□□	I have people around me that I can talk to when I need
	I had no visitors when sick or in hospital	□□□□□	The people around me make an effort to reach out to me when I'm sick

COVID-19, Coronavirus disease of 2019; HVD, Heart valve disease; QOL, Quality of life

It became apparent there were four ways in which features of QOL could be used to generate items. Firstly, a semantic differential could be directly and organically generated by using points given by two participants where one participant reported a negative extreme and the other reported the corresponding positive extreme.

Secondly, a participant could report a single experience of one extreme. This was used to generate one statement, and the opposing statement was generated by the research team to oppose it.

Thirdly, a participant could report an experience that was in the middle of the spectrum between extremes, and so two statements were generated by the research team to form opposing extremes around the participant’s moderate experience.

Finally, participants could report a wide range of experiences that could not be immediately transformed into a single item. In these cases, the experiences were used to generate multiple subtly different items covering similar topics, allowing participant information to be used with the expectation that later phases of testing would identify which item(s) should be retained.

The reader may notice that not all items in 3: Semantic Differential are ‘balanced’. That is, item statement pairs are not truly conceptually opposite; for example, the statement “I feel embarrassed because I need help even in basic activities” states embarrassment as deriving from needing help in tasks, whereas its paired statement, “I have no need to feel embarrassed about my health” states embarrassment as deriving from health status. These concepts are not the same, making the item flawed. A Likert scale only requires a single statement to be generated, and so this discrepancy could not occur—meaning that the researcher generating the statements would not have to consider the difference between the two concepts posed. A semantic differential has the potential to highlight subtle differences between concepts.

Table 4 shows examples where two statements described different concepts, highlighting an issue, which was then corrected by statement wording changes. The final items were assembled into a 64-item questionnaire for further testing.

**Table 4:** Item statement imbalances

Initial statement pair	Issue	Changes made
I'm afraid to leave the house in my current state of health/I feel comfortable about leaving the house in my current state of health	“Afraid” and “comfortable” are not opposites	‘Comfortable’ changed to ‘confident’
Walking takes a lot more energy than it should/Walking is energising	“Takes a lot more energy than it should” and “energising” are not opposites	“Energising” changed to “easy”
I don't have anything to look forward to/I have things to look forward to	Left hand statement has an extreme position whereas the right hand statement has a more moderate position, and therefore needs to be made more extreme	Right statement changed to “I have exciting things to look forward to”
I have to factor in my heart valve disease every time I make decisions or plans/I can ignore my heart valve disease when I make decisions or plans	“Factor in” is not a perfect opposite of “ignore”, and also simpler phrasings are available	‘Factor in” changed to “consider”

### Questionnaire feedback—questionnaire as a whole

In Phase Two, participants completed a form asking what they liked about the full 64-item questionnaire, what they disliked or found confusing, and what they thought was important but was not covered in the questionnaire. Participants found the questionnaire generally good, understandable, and in no need of further changes.

In the section of what participants found disliked or confusing, no participant expressed confusion over the semantic differential format. Only one participant gave specific feedback in this section, and it related to clinical communication rather than the questionnaire content: “A little confusing with some questions as…my problem hasn't been fully discussed or explained by my [general practitioner]” (participant 015).

In the section on what the questionnaire had missed, multiple participants gave specific feedback, including that the questionnaire should have more questions on sleep and life stressors (participant 045), medications and foods they have to avoid (participant 046), the effect of other operations (participant 061), and the effect of COVID-19 restrictions (participant 064). Since these topics did not directly reflect QOL in HVD, they were not added into the questionnaire.

### Questionnaire feedback—individual items

Three items had at least three respondents provide no response, leaving the question blank, across the entire questionnaire: items 20 and 50 had three respondents provide no response, and item 27 had four responders provide no response. This may indicate these items are possibly flawed. If we investigate these items further:

Item 20 consisted of the statements “I’m able to do less than doctors predicted”/“I’m able to do more than doctors predicted”. The participants who did not give a response to the item left the following feedback on the item: “No predictions made”, “Dr hasn’t predicted anything”, “I don’t know what the doctors ‘predicted’”, and “? he didn’t predict”. Participants who completed the item gave similar feedback. Therefore, the item was deemed flawed and was removed from the questionnaire.

Item 50 consisted of the statements, “I'm worried about the consequences I could have if I need an operation for my heart valve disease in the future (such as needing time off work, or pain)”/“I don't have any worries about the consequences of potential future operations for heart valve disease (such as needing time off work, or pain)”. Participants who left the item blank gave the feedback, “this is the doctor’s call”, “N/A”, and “not having an operation”. Participants who completed the item gave different feedback, noting specific features relating to their response, such as, “I worry about recovery time” and “OK – luck may be on my side”. The item was deemed inapplicable to a potentially large subset of the study population and was therefore removed from the questionnaire.

Item 27 consisted of the statements, “I can't keep up with my friends when we do physical activity together”, “I can easily keep up with my friends when we do physical activity together”. Participants who did not complete the item gave the feedback, “N/A”, “not applicable”, “never tried”, and “rest home”. Participants who completed the item gave similar feedback, and the item was removed.

Feedback on other items mostly pertained to clinical features and other details of the participants’ experiences, not the format. Some items, however, had feedback relating to their format and the phrasing of their statements.

Item five, which consisted of the statements, “I get short of breath even when I'm sitting quietly”/“I only get short of breath when I exercise very hard” had two respondents leave the item blank, saying “never short of breath” and “don’t exercise”. Feedback from respondents who completed the item included “I do get short of breath when I’m walking, but not when I’m sitting quietly. I found this question ambiguous—is it ONLY referring to when you are sitting quietly?” Another participant crossed out the “only” and “very hard” on the right-hand statement. The item, therefore, did not balance; a statement had to be adjusted. Interestingly, while statements containing multiple concepts are generally flawed, this question required an addition: the right-hand statement was changed to “I only get short of breath when I exercise very hard. OR I never get short of breath.”

Item six, which consisted of the statements, “My symptoms are unpredictable. I don't know when they will occur” /My symptoms are predictable. I know when they will occur” had similar feedback and also required an addition; the right-hand statement was changed to “My symptoms are predictable. I know when they will occur. OR I don't get symptoms”.

Item 42 consisted of the statements, “I don't trust doctors/the healthcare system”/“I trust doctors/the healthcare system”. A respondent did not leave a response to the item, but left the comment, “I trust my doctor but not too much the healthcare system”. Due to the two concepts within this single item, respondents can have different levels of agreement for each concept, and so one concept was removed from the item; the item was changed to “I don’t trust the healthcare system”/“I trust the healthcare system”.

## Discussion

Accurate measurement of QOL in chronic disease is an increasingly important aim in our aging population [20], with QOL in cardiac diseases being contributed to by a wide range of inter-relating factors [21]. There are numerous questionnaires to measure QOL, but standard practice is dominated by Likert scales [22], or different questionnaires with a very similar format [23], and alternative formats are rarely considered, despite limitations of current tools to capture the dynamics of QOL in a lifelong disease [24]. This article describes the development of a semantic differential scale for use in the health sciences and initial feedback related to this scale. It showed that a semantic differential scale, which is generally considered to be more difficult to write and to respond to than a Likert, can improve the writing of items, and in this case was generally liked by respondents. In addition, it may better capture the perspectives of respondents [25].

Respondent burden is a key consideration in questionnaire generation, as questionnaires that impose more cognitive burden on respondents are more likely to not be completed, or to cause acquiescence bias in respondents [26] (acquiescence bias being when, due to mental fatigue, a respondent selects one level of response throughout a questionnaire without due consideration as to whether this response reflects their experience relative to the questionnaire items). A semantic differential has twice the number of statements as a Likert, and demands the respondents perform a more complex mental calculation to determine their relative position between items, and can therefore be expected to increase respondent burden and thus acquiescence bias [27].

However, our experience found this may not be the case in practice. When the questionnaire was pilot tested, response rates were sufficient even though the questionnaire for pilot testing was 64 items long: 41% of approached individuals completed the mailed questionnaire, which is approximately the average response rate for mail-only physician-sent surveys [28].

Some items had too many respondents leave the item blank, indicating that these items were unsuitable, but according to respondents’ feedback, this was likely due to item content, not the item’s format;none of the respondents noted that the format of the items was an issue. Formatting an item to have multiple concepts within the item can lead to confounding and error [29], but our experience with the semantic differential found that this format made it easier to identify when this had occurred.

Some participants noted in their feedback that the semantic differential format required more effort; participant 65 noted, “being two options made me think more”, and they considered the questionnaire “good” overall. This, however, rather than confirming acquiescence bias in semantic differential questionnaires, suggests the opposite: if respondents put in more effort, the risk of acquiescence bias may be reduced. Modern computational tools such as eye-tracking could provide qualitative assessment on this issue [30].

Moreover, the assumption that a semantic differential necessarily requires more effort may be incorrect; participant 82 noted, “Easy scale between negative and positive to work out where you fit on the scale”. It is possible that rating a position between two points is, in some cases, a simpler mental calculation than rating position relative to a single, open-ended Likert statement. Current literature, however, cannot confirm this; more head-to-head comparisons between Likert and semantic differential questionnaires are required.

There is little direct head-to-head comparison of Likert and Semantic differential formats in health research, and while the semantic differential is occasionally used in this discipline, the rationale of its use as compared to the more common Likert is rarely discussed. One existing study, however, compared the formats and found that the Likert had better construct validity [31]. However, another study found that the semantic differential had better-fitting factor structure, and the authors also concluded that the semantic differential may reduce acquiescence bias [32].

Outside of health research, some marketing analysts prefer the semantic differential due to reduced framing bias [33], which may be of particular use in reducing bias when measuring across different cultures [34]. Evidence suggests that framing and acquiescence bias are linked [35], which again suggests that future research be conducted to compare the semantic differential to other more commonly-used formats. It is also becoming increasingly apparent, as computational analyses improve, that mapping the underlying concepts contributing to QOL is key for accurate assessment of QOL [36]. The semantic differential format, in our case, forced us to clearly identify exactly what our item was asking respondents about (in contrast to an open-ended and thus less clearly defined Likert item statement), which may aid researchers in qualitatively generating more robust questionnaires that can be quantitatively tested.

### Limitations

This is a use-case study that suggests further investigation into the use of semantic differentials in clinical research. The VALVQ development programme initially planned to use a Likert scale, similar to other health related QOL instruments; this project was not designed to compare questionnaire formats, and it cannot be used as a comparison between semantic differentials and Likerts, or prove the superiority of either. Like most clinician-researchers, we simply were not aware of the existence of the semantic differential scale in this setting at the project’s outset. However, at the time of item development, we trialled the semantic differential format internally and found it to be worth using based on both the literature from other disciplines [37, 38] and the readability of the questionnaire. We hope our examples of how qualitative data can be transformed into bipolar items may aid future research by giving researchers a starting point for more in-depth analyses and comparisons between the semantic differential and Likert formats, and by simply raising awareness of the semantic differential format to clinician-researchers.

## Conclusion

Semantic differential scales can be developed for assessment of health-related QOL, and can be considered as an alternative to Likert scales in this setting. Future QOL research should compare the accuracy and acceptability of Likert and semantic differential scales in health research.

## Supplementary Information

Below is the link to the electronic supplementary material.

Appendix

## Data Availability

The data supporting this article cannot be shared publicly due to the conditions of ethical approval.
