# Supplementary material for: The semantic differential questionnaire format warrants consideration for use in healthcare settings
Source: Qual Life Res. 2026 Mar 8;35(4):94. doi: 10.1007/s11136-026-04198-9 (PMC12968091; doi:10.1007/s11136-026-04198-9)
Supplement: Supplementary file 1 — Appendix [file 11136_2026_4198_MOESM1_ESM.docx]

**Appendix to Pons et al, “ The semantic differential questionnaire format warrants consideration for use in healthcare settings”**

*Appendix Table One: COREQ Checklist*

| **No** | **Item** | **Guide questions/description** |
| --- | --- | --- |
| **Domain 1: Research team and reflexivity** |  |  |
| Personal Characteristics |  |  |
| 1 | Interviewer/facilitator | Ariel Pons |
| 2 | Credentials | PhD student |
| 3 | Occupation | Medical student |
| 4 | Gender | Female |
| 5 | Experience and training | Online course: Focus Groups (Coursera) Medical education on interviewing as part of MBChB |
| Relationship with participants |  |  |
| 6 | Relationship established | Participants were sent information sheet and consent form before participation |
| 7 | Participant knowledge of the interviewer | Participants knew the interviewer was a PhD student, that the results would be used for papers and also the student’s thesis, and the aims of the project |
| 8 | Interviewer characteristics | No characteristics were reported about the interviewer |
| **Domain 2: study design** |  |  |
| Theoretical framework |  |  |
| 9 | Methodological orientation and Theory | Phenomenology |
| Participant selection |  |  |
| 10 | Sampling | Consecutive convenient |
| 11 | Method of approach | Telephone, cell phone, and post |
| 12 | Sample size | Phase one: 42; phase two 46; phase three 290 |
| 13 | Non-participation | Phase one: 27% participation rate. Nonparticipants mostly could not be contacted (80%); 11% declined participation and 7% lost to followup.  Phase two: 68% participation rate. Nonparticipant reason not assessed.  Phase three: 46% participation rate. Nonparticipant reason not assessed. |
| Setting |  |  |
| 14 | Setting of data collection | Postgraduate office, Department of Medicine, Dunedin Hospital |
| 15 | Presence of non-participants | Participant’s family members were sometimes present during interviews |
| 16 | Description of sample | Samples collected from October 2019 to December 2021. Roughly half female and ethnicity mostly NZ European. Participants often elderly and with comorbid conditions. |
| Data collection |  |  |
| 17 | Interview guide | Interview was semi-structured |
| 18 | Repeat interviews | Repeat interviews not collected. |
| 19 | Audio/visual recording | Audio recording was used to generate transcripts |
| 20 | Field notes | Field notes not made |
| 21 | Duration | Duration of interviews was 15 minutes to one hour. |
| 22 | Data saturation | Data saturation was discussed with the research team. |
| 23 | Transcripts returned | Transcripts were not returned to participants. |
| **Domain 3: analysis and findings** |  |  |
| Data analysis |  |  |
| 24 | Number of data coders | There was one primary data coder, with assistance from three others |
| 25 | Description of the coding tree | Coding tree not used. Data structure specified in protocol. |
| 26 | Derivation of themes | Themes were derived from data. |
| 27 | Software | Data and thematic groupings were stored in excel. |
| 28 | Participant checking | Participants gave feedback in phase two, pilot testing. |
| Reporting |  |  |
| 29 | Quotations presented | Participant quotations were included. Participants’ study numbers were sometimes included but sometimes not for ease of reading. |
| 30 | Data and findings consistent | Data and findings are consistent. |
| 31 | Clarity of major themes | Major themes were presented in text. |
| 32 | Clarity of minor themes | Themes are described. |

*Appendix Table Two: Interview Structure*

| Initial structure  (Determined by first four interviews) | Final structure |
| --- | --- |
| Participant’s definition of QOL/wellbeing | Participant’s definition of QOL/wellbeing |
| What a ‘good’ and a ‘bad’ day consisted of | Participant’s current QOL |
| How a ‘bad’ day could become a ‘good’ one | Progression of QOL over time |
| Checklist of items volunteered by other participants as important without the interviewer specifically asking, and whether the current participant thought they were important: Family, physical independence, pain, and challenges. | Checklist of items volunteered by other participants: Fatigue, pain playing a defining role in life, medications, and experiences with doctors and the healthcare system |
| Effect of HVD on QOL. If no significant effects had been noted to this point in the interview, the participant was asked to go through a typical day, and note the impacts of HVD at each point. |  |
| Progression of QOL over time |  |

*Appendix Table Three: Pilot-tested Items, Distributions, and Respondent Feedback*

| Item | Distribution | Subject Identifier: Comments | Comments when left blank |
| --- | --- | --- | --- |
| 1: I'm unhappy with my level of physical activity - I'm happy with my level of physical activity | 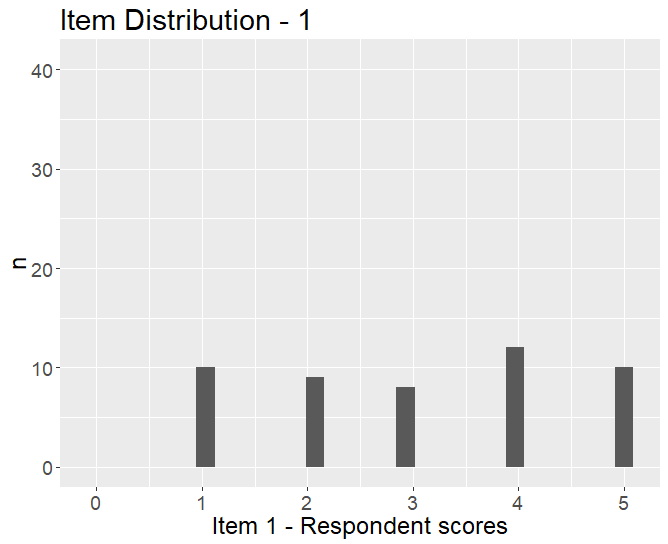 | p2_19: Noted in question at end of 'anything you particularly liked about this questionnaire' that they specifically liked this question.  p2_000: “I tire quickly!” (Score: 3)  p2_031 (VR): “I have started to go swimming, having a little difficulty” (Score: 3)  p2_032 (VR): “not HVD related” (Score: 2) |  |
| 2: I'm unable to do the things I enjoy - I'm able to do the things I enjoy | 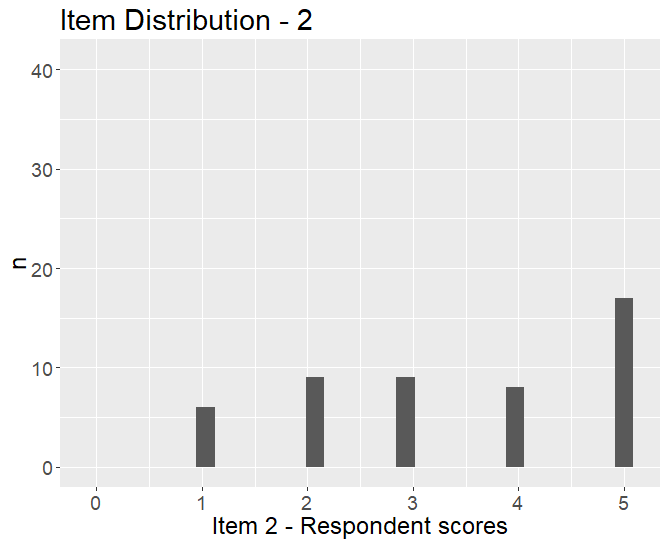 | p2_000: “Gardening, baking” (Score: 3)  p2_011 (MR): “going out with friends, dancing, walking my dog, running around with my kids” (Score: 1) |  |
| 3: I don’t eat well – I eat well | 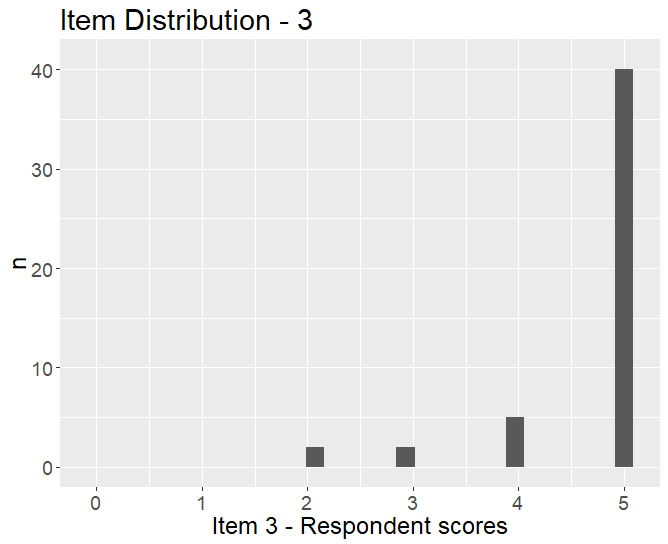 | p2_011 (MR): “Sometimes have reflux or [???] fluid and don’t feel hungry” (Score: 2)  p2_027 (VR): “Gained approx 10 kilos since op” (Score: 5)  p2_061 (VR): “too well” (Score: 5) |  |
| 4: I am always in pain – I am never in pain | 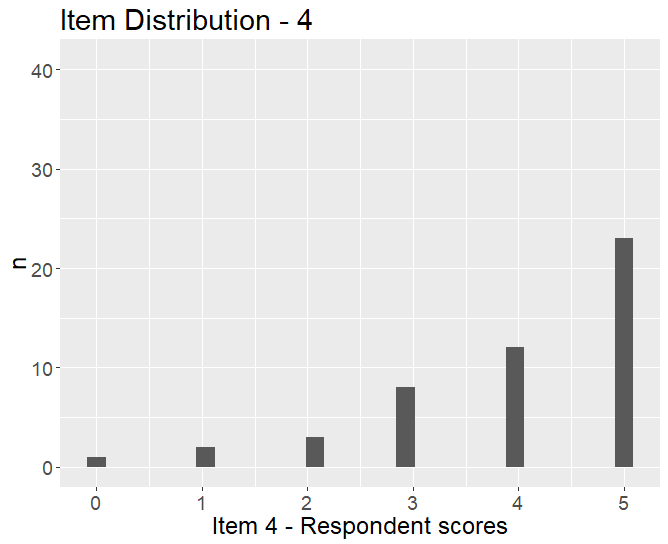 | p2_000: “Either back, legs, or hands” (Score: 3)  p2_011 (MR): “often in pain, reflux or skin, joints after sitting for a while” (Score: 2)  p2_031 (VR): “In the chest, I suppose it's because of the repeat operation I had” (Score: 3)  p2_080 (MR): “pain is kept under control with medications” (Score: 3) |  |
| 5: I get short of breath even when I'm sitting quietly - I only get short of breath when I exercise very hard | 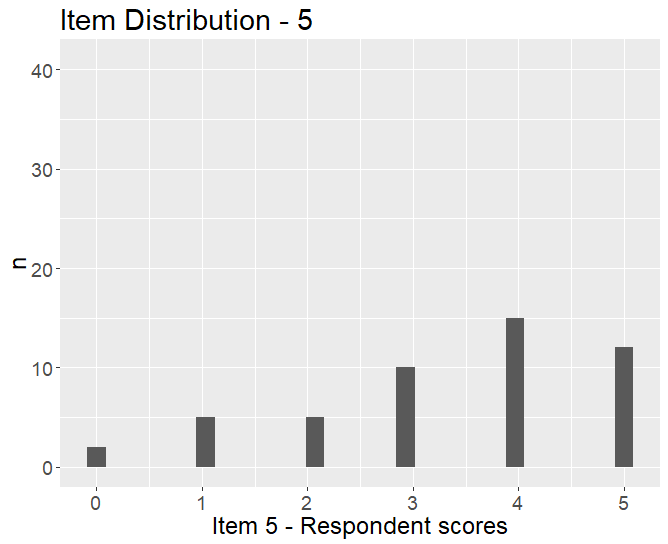 | p2_011 (MR): “when I bend over, moving around, when I’m holding fluid” (Score: 4)  p2_032 (VR): [Had crossed out the 'only' and 'very hard' of right hand statement] “Also when I experience difficulty with medications” (Score: 3)  p2_040 (MR): “Question 5: I do get short of breath when I'm walking, but not when I'm sitting quietly. I found this question ambiguous - is it ONLY referring to when you are sitting quietly?” | p2_003 (AS): “Never short of breath”  p2_015 (MR): “don't exercise” |
| 6: My symptoms are unpredictable. I don't know when they will occur. - My symptoms are predictable. I know when they will occur. | 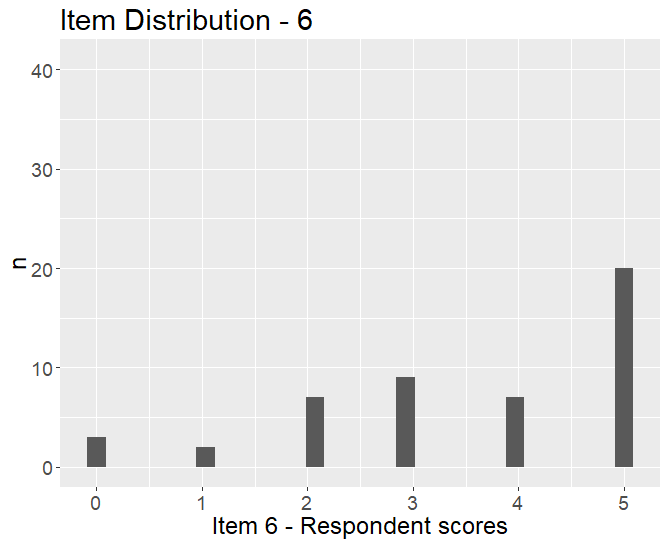 | p2_000: “When I try to walk a longer distance.” (Score: 4)  p2_007 (AS): “Governed by my back as lumbar vertebrae is 'smashed'” (Score: 3)  p2_013 (MR): “usually” (Score: 5)  p2_031 VR): “I feel the chest wound has not yet healed properly” (Score: 4)  p2_045 (MR): “apart from breathlessness” (Score: 5) | p2_003 (AS): “No symptoms apart from lack of horsepower”  p2_015 (MR): “don't know about symptoms”  p2_027 (VR): “no symptoms” |
| 7: I don't have the time to take breaks, even when I need them to relieve tiredness or my symptoms - I have the time to take breaks when I need to relieve tiredness or my symptoms | 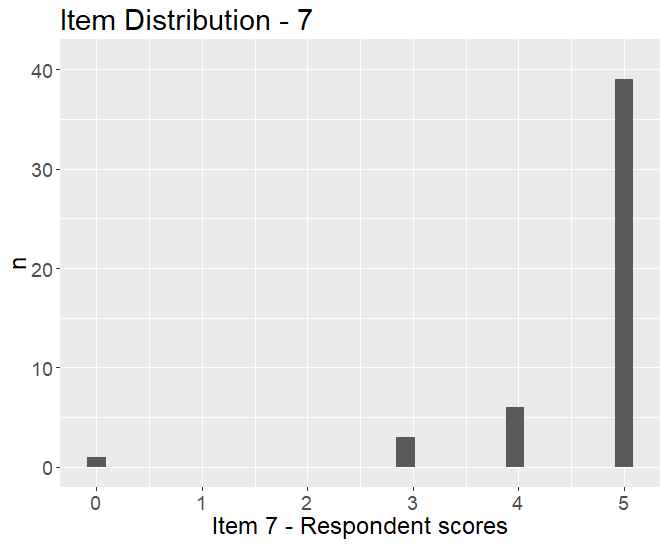 | p2_000: “Sometimes I try to take a break” (Score: 3)  p2_009 (MR): “I am retired. so have plenty of time to take breaks.” (Score: 5)  p2_011 (MR): “I struggle with time getting ready before appointment” (Score: 4)  p2_027 (VR): “retired” (Score: 5)  p2_031 (VR): “I used to sleep in the afternoon but not as often as used to” (Score: 5)  p2_080 MR): “82 and retired - lots of time” (Score: 5) |  |
| 8: I'm frustrated by my lack of energy - I'm happy with how much energy I have | 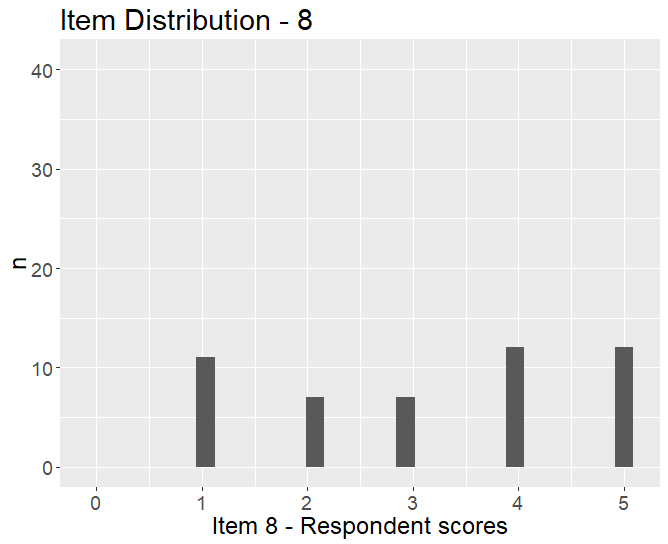 | p2_000: “Still not able to hang laundry on the line” (Score: 1)  p2_00B: “I am 86 so I am happy with my energy level” (Score: 5)  p2_007: “I'm physically restricted” (Score: 3)  p2_031: “Energy levels are up, or maybe I am just getting used to it” (Score: 4) |  |
| 9: My achievements are only very small things - I can achieve big things | 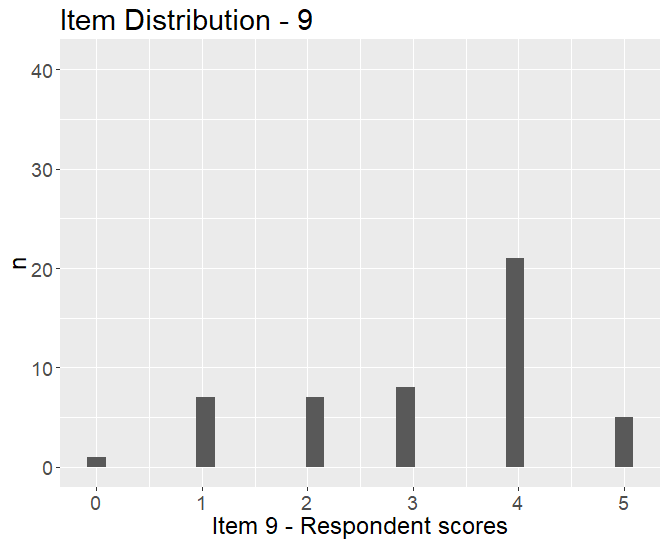 | p2_000: “Takes a long time to dress myself” (Score: 2)  p2_00B: “moderate” (Score: 4)  p2_013 (MR): “for my age” (Score: 5)  p2_031 (VR): “I feel I am still pretty strong” (Score: 4) | p2_045 (MR): “? Too subjective value?” |
| 10: I'm afraid to leave the house in my current state of health - I feel comfortable about leaving the house in my current state of health | 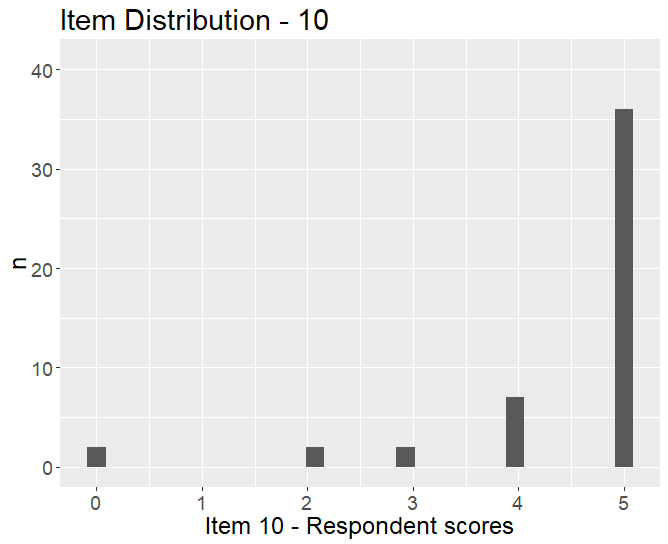 | p2_009 (MR): “Take the car, even for short distances” (score: 5)  p2_025 (VR): “Only go out with my husband assisting me. Have walking stick or walking frame” (score 5)  p2_026 (RHD): “A family member takes me out.” (score: 3) | p2_100 (VR): “Now in rest home” |
| 11: My heart valve disease disrupts my sleep - My heart valve disease doesn't disrupt my sleep | 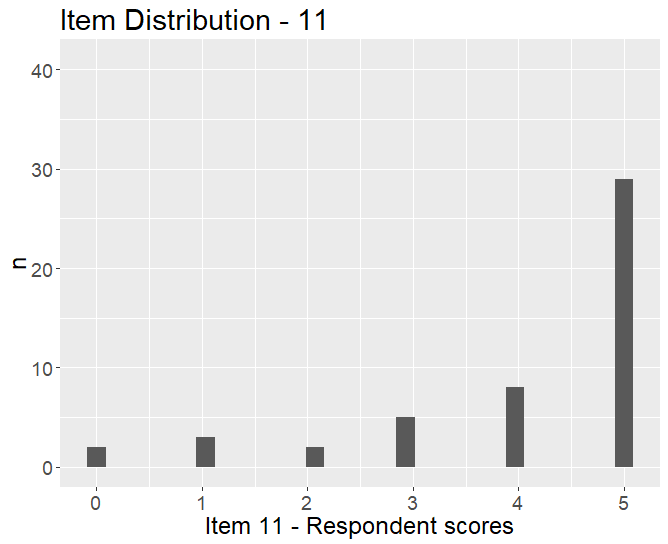 | p2_000: “Restless, up all the time” (Score: 1)  p2_007 (AS): “I've had a heart valve replaced” (Score: 5)  p2_011 (MR): “bathroom - fluid tabs” (Score: 2)  p2_025 (VR): “Dreams, nightmares, breathlessness” (Score: 2)  p2_031 (VR): “It's ok if I keep diabetes in control” (Score: 5)  p2_08 (MR): “occasionally wake up with chest pain” (Score: 4) | p2_027 (VR): “unsure as I've always had sleep problems”  p2_032 (VR): “the effect of medications severely disrupts my sleep” |
| 12: Walking takes a lot more energy than it should - Walking is energising | 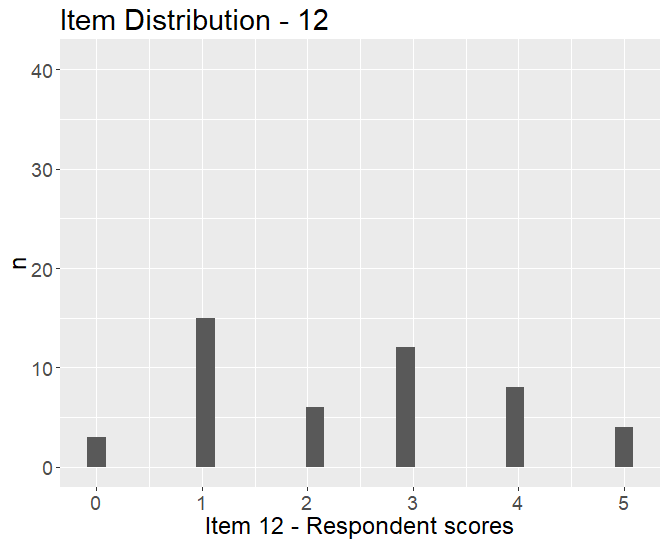 | p2_000: “Slow, short distances only” (Score: 2)  p2_00B: “Stiffness in my lower back? Difficult to get up and down - nothing to do with my heart” (Score: 3)  p2_025 (VR): “Get breathless, brainfog after 1 hr” (Score: 2)  p2_031 (VR): “I mainly go aqua jogging” (Score: 4) | p2_015 (MR): “I don't do walking. I garden.”  p2_028 (VR): “not walking but love on my bike” |
| 13: My symptoms scare me - My symptoms don’t scare me | 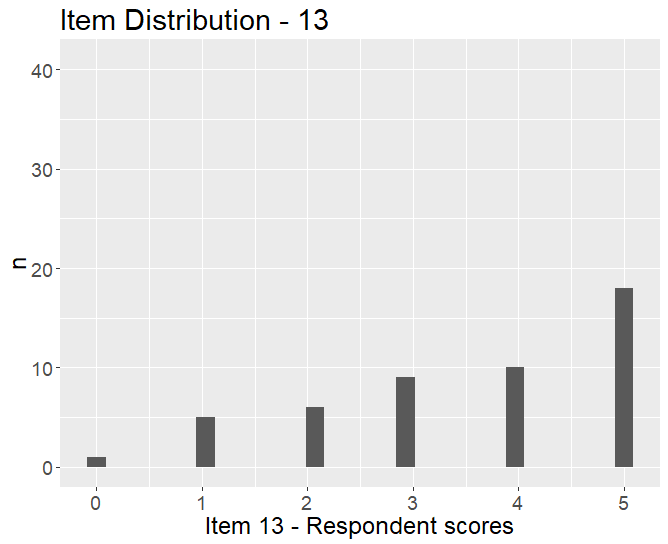 | p2_000: “They frustrate me!” (Score: 2)  p2_011 (MR): “breathless, dizziness, when heart rate is high” (Score: 2)  p2_026 RHD): “suffocating.” (Score: 3)  p2_032 (VR): “from medication only” (Score: 1)  p2_082 (VR): “Don’t have symptoms” (Score: 5) | p2_015: “?” |
| 14: It is unsafe for me to do physical activity - It is safe for me to do physical activity | 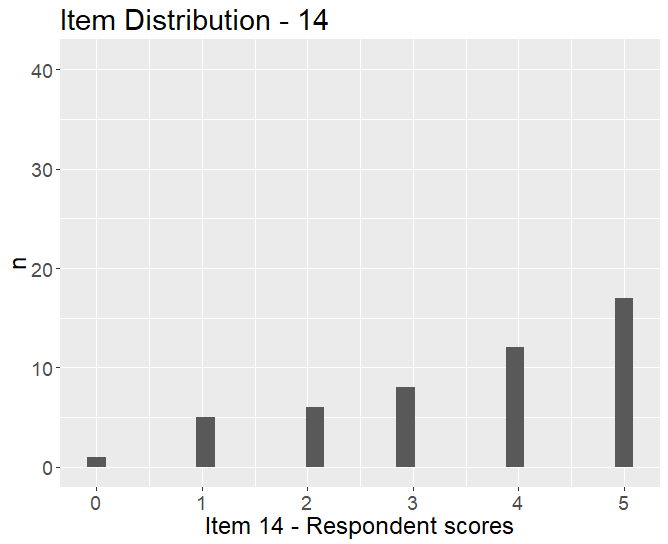 | p2_000: “Sometimes I don't balance and my legs give up” (Score: 3)  p2_007 (AS): “My back restricts activities” (Score: 2)  p2_026 (RHD): “feeling more frail” (Score: 2)  p2_080 (MR): “depends on the level” (Score: 3) |  |
| 15: I'm no longer able to do as much physical activity as I used to - I'm able to do more physical activity than I used to | 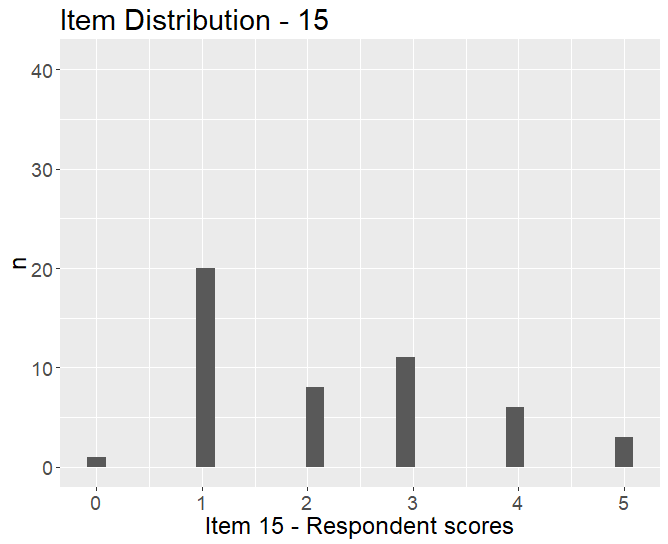 | p2_003 (AS): “Nearly 85 after all!” (Score: 3)  p2_007 (AS): “Restricted by my back” (Score: 1)  p2_011 (MR): “was able to do house work, shopping, socialising, I use to work 25/30 hours a week as a vet nurse and walk 4.8K to work” (Score: 1)  p2_025 VR): “I chose my physical activity to the things I can do” (score: 3) | p2_028 (VR): “new hip getting there” |
| 16: My current state of health is worse than I expected - My current state of health is better than I expected | 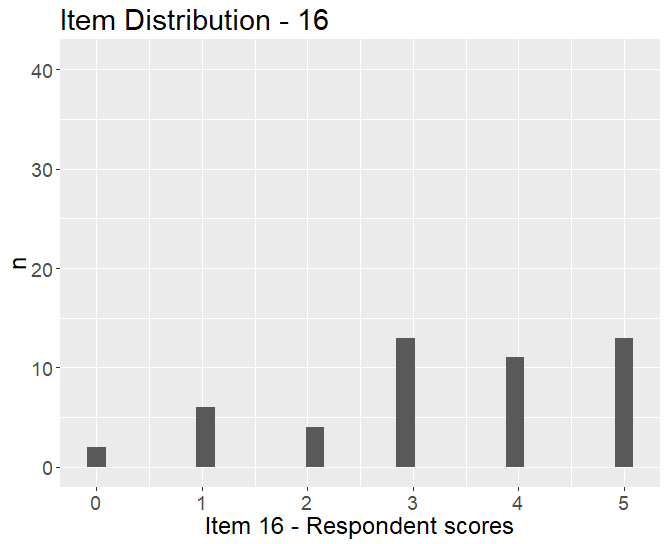 | p2_003 (AS): “OK” (Score: 4)  p2_011 (MR): “I didn't think I would get sick till later in life” (Score: 1)  p2_109 (MR): Not sure what relating to. Question asked if health better or worse than expected - what was that in relation to? Age, disease? | p2_045 (MR): “? meaning?”  p2_109: “Not sure what is meant by this question” |
| 17: Exercise makes me feel worse - Exercise makes me feel better | 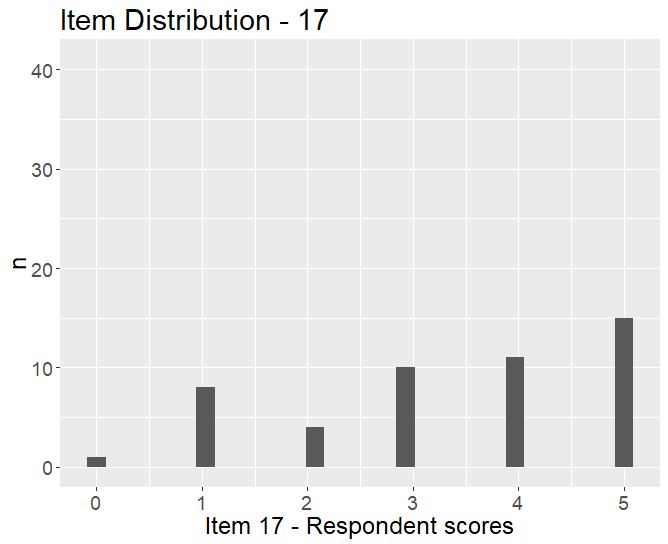 | p2_026 (RHD): “shortness of breath” (Score: 1)  p2_040 (MR): “ Exercise makes me feel better OVERALL afterwards, but breathless when doing it” (Score: 5) | p2_025 (VR): “Makes me very fatigued, but do gentle exercise” |
| 18: I can't look after myself - I can look after myself | 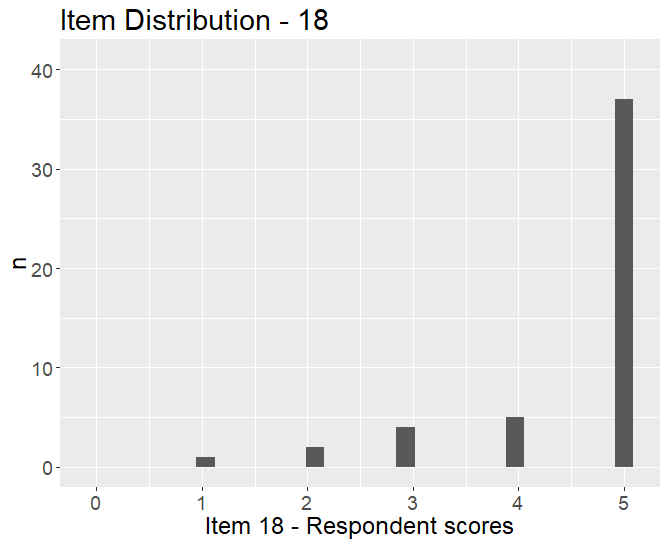 | p2_000: “Some house chores I can't manage” (Score: 4)  p2_007 (AS): “My husband assists me wherever necessary” (Score: 2)  p2_011 (MR): “struggle - housework, cooking, shopping - lucky to have my husband” (Score: 3)  p2_025 (VR): “My husband and I share the load” (score: 3)  p2_031 (VR): “My wife keeps a good eye on me as well as daughter and sisters” (Score: 4) |  |
| 19: I can't concentrate for long periods - I can concentrate for long periods | 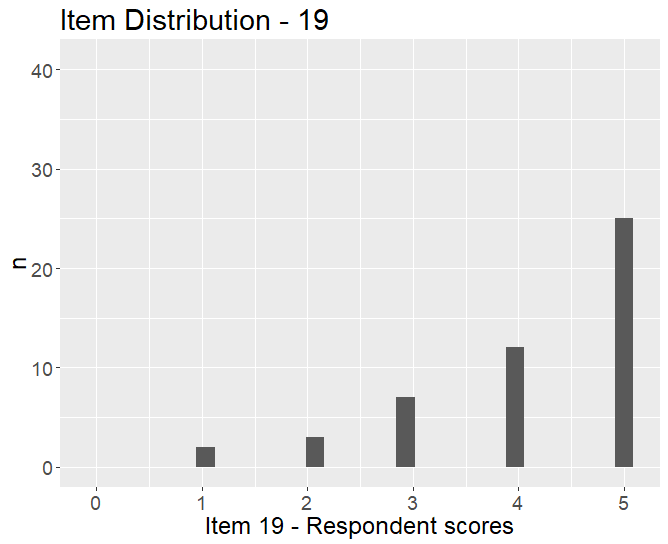 | p2_007 (AS): “MVA 1997 Multiple injuries involving head – concussion” (Score: 3)  p2_011 (MR): “hard to read, paperwork,” (Score: 3)  p2_025 (VR): “Brain fog, exhausting” (Score: 2)  p2_026 (RHD): “having short term memory loss” (Score: 3)  p2_031 (VR): “On most things if I'm interested, eg history/politics, etc” (Score: 4) |  |
| 20: I'm able to do less than doctors predicted - I'm able to do more than doctors predicted | 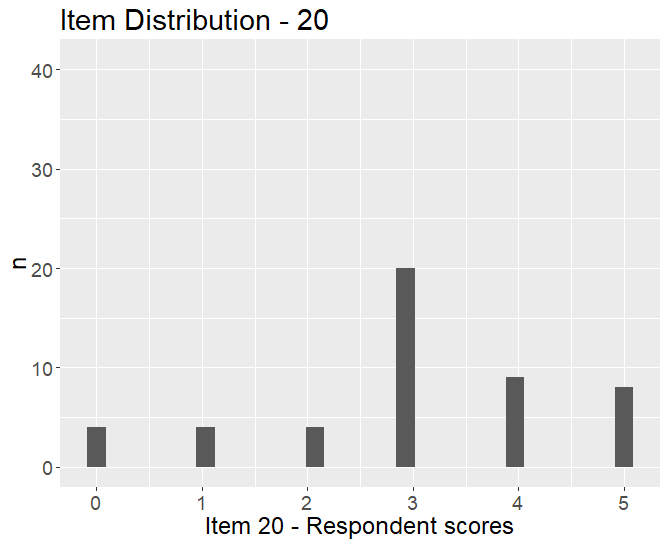 | p2_011 (MR): “some days are flatter than others” (Score: 2)  p2_027 (VR): “I don't know what they predicted” (Score: 3)  p2_031 (VR): “It took me longer to get walking faster than I was told” (Score: 4)  p2_070 (AS): “I don't recall receiving advice as to this question” (Score: 3)  p2_082 (VR): “Doctors didn’t predict” (Score: 5) | p2_003 (AS): “No predictions made”  p2_015 (MR): “Dr hasn’t predicted anything”  p2_040 (MR): “I don't know what the doctors "predicted"”  p2_045 (MR): “? he didn't predict” |
| 21: I'm unable to do as much as other people my age - I'm able to do more than other people my age | 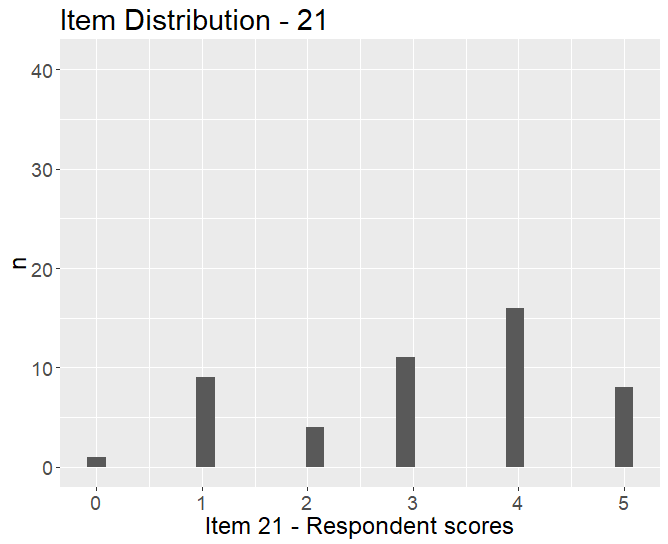 | p2_007 (AS): “Ongoing problems from MVA 1997” (Score: 3)  p2_011 (MR): “sad when I see friends” (Score: 1)  p2_031 (VR): “I am still amazed at what older men can do” (Score: 4) |  |
| 22: I need help for basic, everyday tasks - I can do basic, everyday tasks by myself | 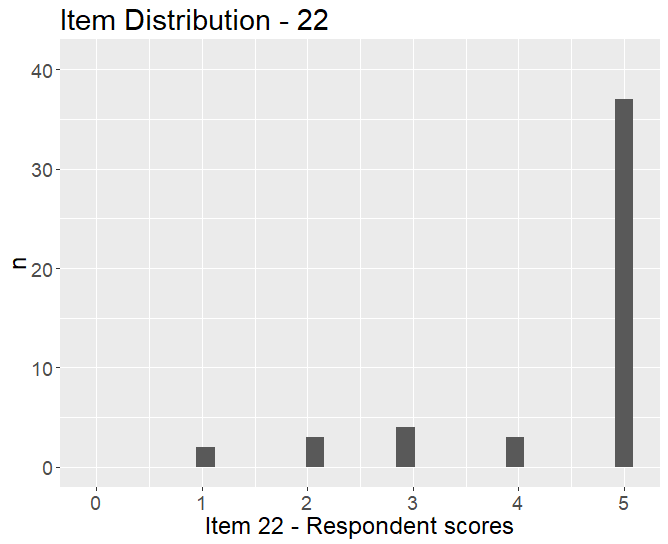 | p2_000: “Cleaning bathroom, toilet, vacuum” (Score: 2)  p2_011 (MR): “house work, going to GP” (Score: 2)  p2_031 (VR): “I find I'm not as good with tech things” (Score: 3) |  |
| 23: I'm unable to work / do projects /do chores - I'm able to work / do projects /do chores | 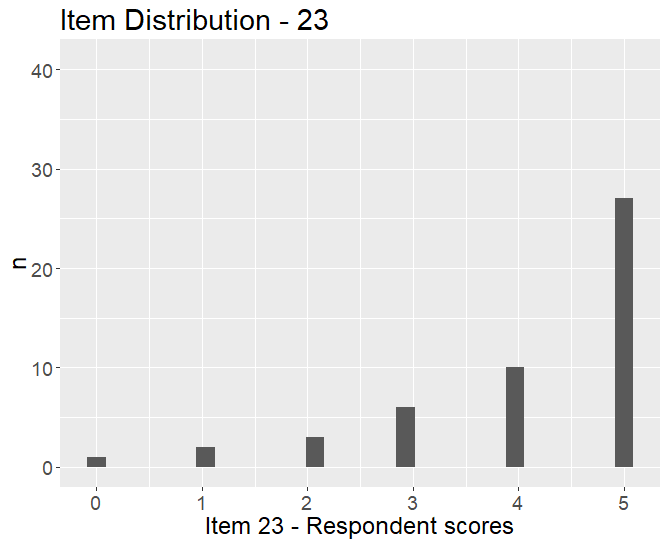 | p2_009 (MR): “I have home help. 1 hr a week for the heavy work” (Score: 3)  p2_011 (MR): “Have not worked in 2 years, hard to vacuum, scrub walls/bath” (Score: 2)  p2_025 (VR): “light gardening, sewing” (Score: 2)  p2_031 (VR): “I'm good at repairs and maintenance” (Score: 5) | p2_000: “Retired” |
| 24: I don't enjoy my work / projects / chores - I enjoy my work / projects / chores | 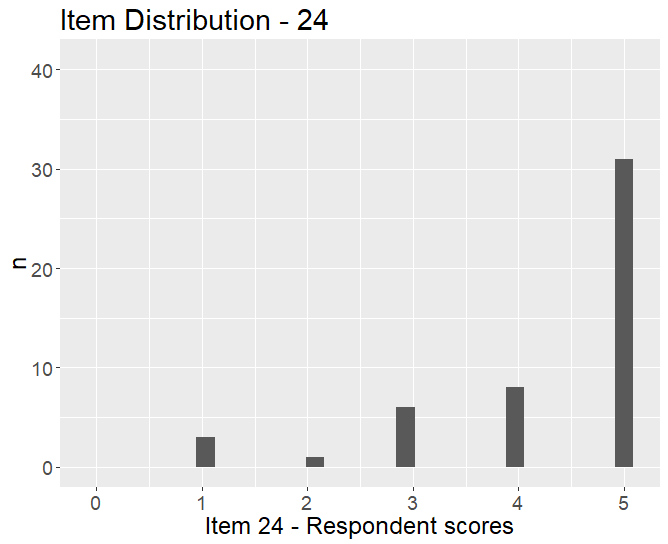 | p2_009 (AS): “Show me one person who likes doing dishes!” (Score: 3)  p2_011 (MR): “love crafting/steampunk, felting, sewing, art” (Score: 4)  P2_031 (VR): “I like landscaping/gardening/painting” (Score: 5) |  |
| 25: Doctors don't listen to what I tell them - Doctors listen to what I tell them | 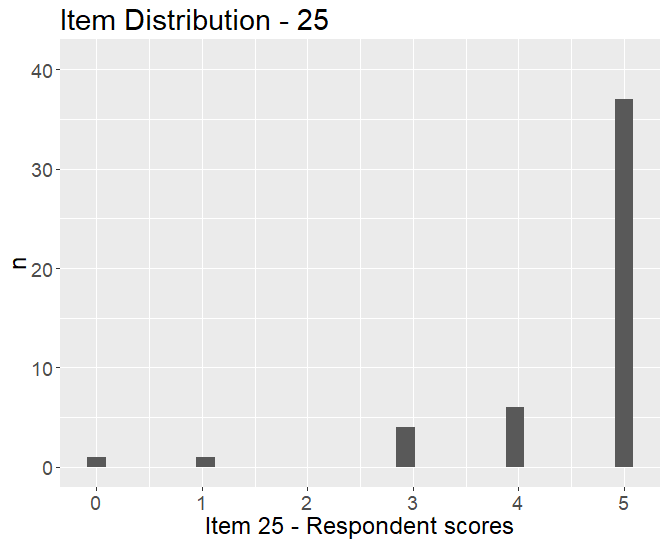 | p2_000: “An excellent Dr.” (Score: 5)  p2_009 (MR): “Most of the time” (Score: 3)  p2_011 (MR): "All my doctors are amazing” (Score: 5)  p2_027 (VR): “haven't needed to see a doctor in ages” (Score: 5) | p2_015 (MR): “I haven't really talked/discussed about problem” |
| 26: I have no motivation - I have lots of motivation | 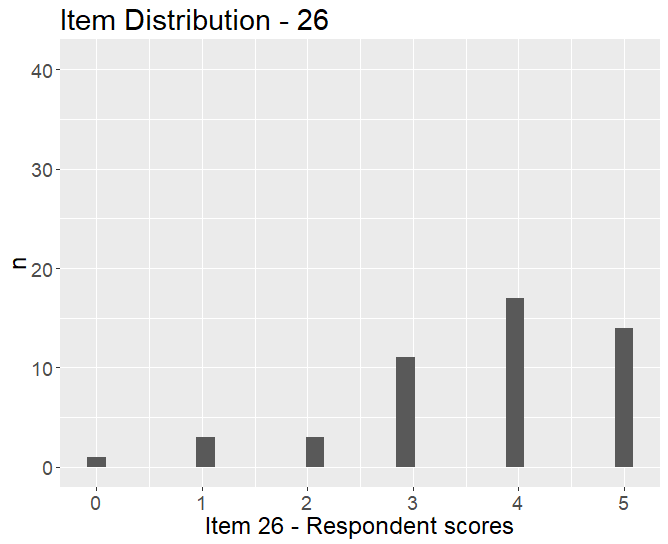 | p2_000: “Some things I am happy to do myself” (Score: 4)  p2_011 (MR): “Some days I sleep, take meds, eat” (Score: 1)  p2_025 (VR): “varies” (Score: 3)  p2_031 (VR): “Looking forward to getting the new campervan we have ordered” (Score: 4)  p2_080 (MR): “? odd question for older person” (Score: 3) |  |
| 27: I can't keep up with my friends when we do physical activity together - I can easily keep up with my friends when we do physical activity together | 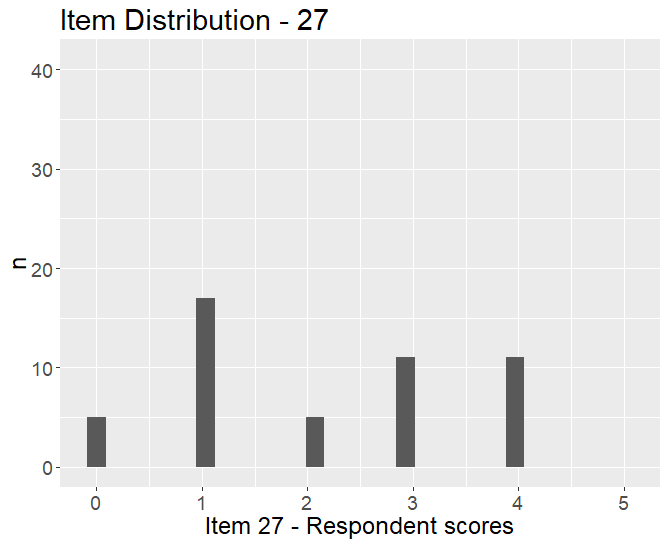 | p2_011 (MR): “I walk so slow” (Score: 1)  p2_019 (RHD): “Do little physical activity with friends” (Score: 2)  p2_028 (VR): “but try” (Score: 1)  p2_031 (VR): “I'm getting better” (Score: 3)  p2_032 (VR): “N/A” (Score: 3) | p2_000: “N/A”  p2_013 (MR): “not applicable”  p2_015 (MR): “never tired.”  p2_089 (VR): “N/A”  p2_100 (VR): “Rest home” |
| 28: I'm stuck at home - I can go wherever I like | 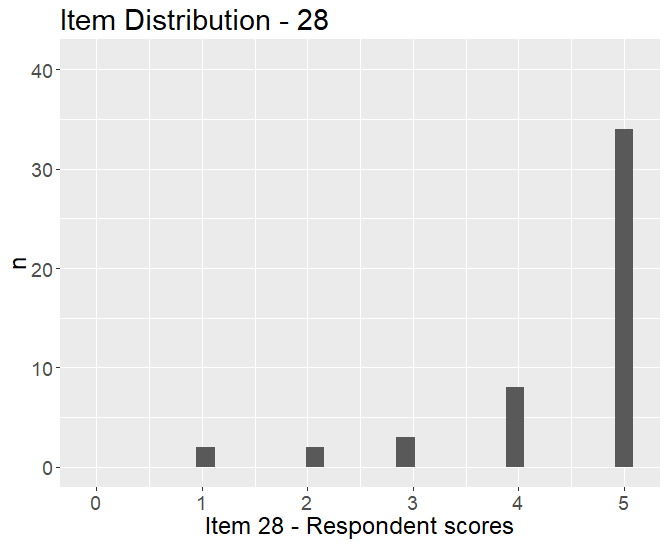 | p2_000: “For now my family drives me” (Score: 3)  p2_007 (AS): “Unable to walk on rough ground” (Score: 4)  p2_009 (MR): “I can drive my car” (Score: 5)  p2_011 (MR): “Hardly leave the house” (Score: 1)  p2_026 (RHD): “I now rely on family to take me out” (Score: 3)  p2_080 (MR): “still drive” (Score: 4) |  |
| 29: I'm not able to be involved in the community activities that I want to do - able to be involved in the community activities that I want to do | 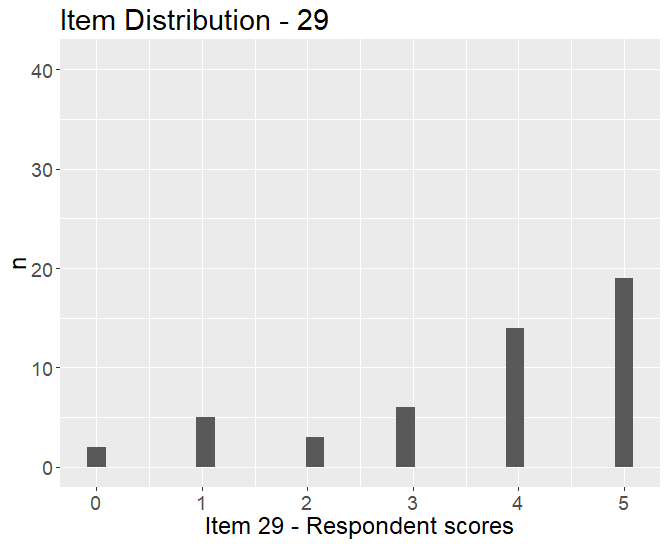 | p2_000: “If I can get someone to take me I'll go” (Score: 3)  p2_007 (AS): “I lead an Age Concern Falls Prevention Class weekly. Co-ordinate grandparent reading group - Concord school” (Score: 4)  p2_009 (MR): “Can't do strenuous activities” (Score: 3)  p2_011 (MR): “Community work, steampunk festival community, school fundraising” (Score: 1)  p2_031 (VR): “Had enough of committees” (Score: 4) | p2_027 (VR): “not involved in any”  p2_089 (VR): “N/A” |
| 30: I'm not able to do as much as I used to - I'm able to do more than I used to do | 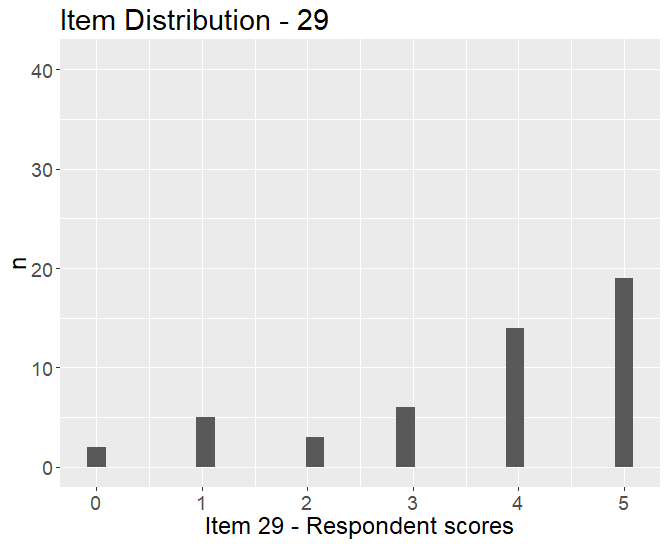 | p2_000: “Tired and slow” (Score: 1)  p2_003 (AS): “Age I guess. 9 holes now instead of 18” (Score: 3)  p2_011 (MR): “Work, walking my dog, cleaning etc - going to parties, birthdays” (Score: 1)  p2_026 (RHD): “Still mostly independent at home” (Score: 2)  p2_031 (VR): “I'm happy with what I can do” (Score: 3) |  |
| 31: I have to travel a long way to get the medical care I need - The medical care I need is provided in my community | 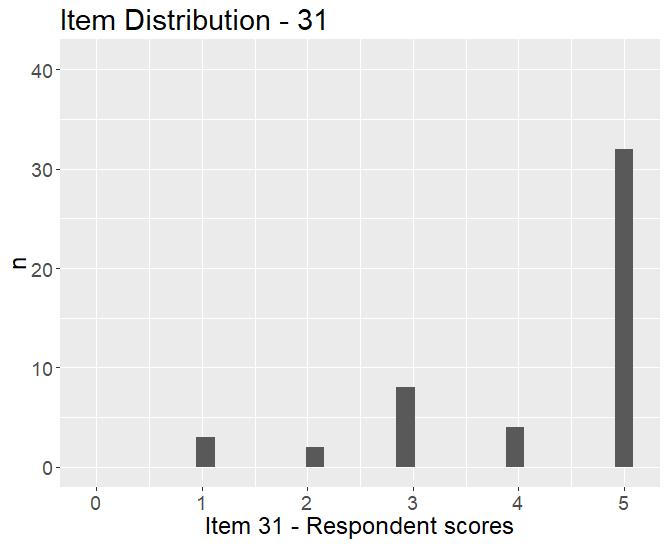 | p2_000: “Some medical problems” (Score: 4)  p2_011 (MR): “1.40min to dunedin airport/hospital, half a day travel to auckland. Travel coordinator is awesome.” (Score: 1)  p2_019 (RHD): “20 K” (Score: 3)  p2_025 (VR): “Cardiologist 1.30 hr away. GP community” (Score: 3)  p2_027 (VR): “35kms” (Score: 2)  p2_031 (VR): “Very good” (Score: 5) |  |
| 32: I've had to let people down - People can rely on me | 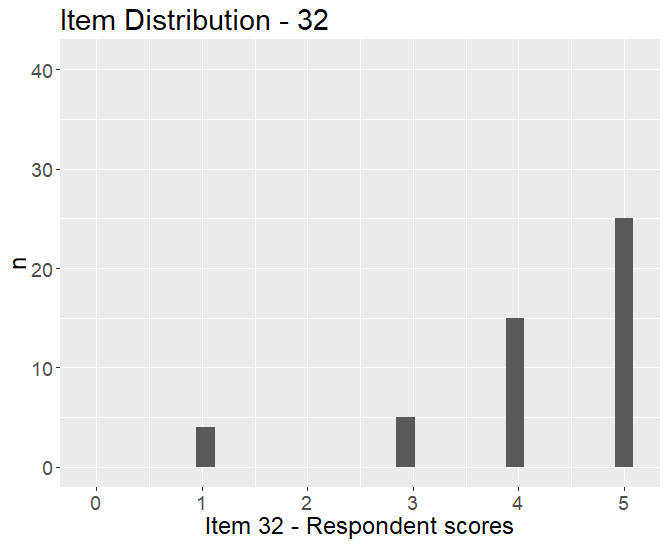 | p2_000: “Miss meetings and mass sometimes” (Score: 1)  p2_011: “Going to events, helping with projects, fundraising” (Score: 1) |  |
| 33: My doctors speak my first language - My doctors speak a language I find difficult to understand | 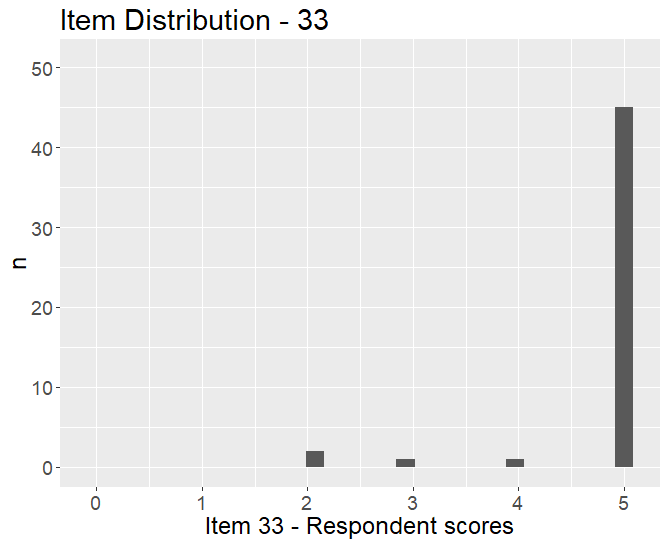 | p2_012: “my first language is Swiss German” |  |
| 34: I don't have anything to look forward to - I have things to look forward to | 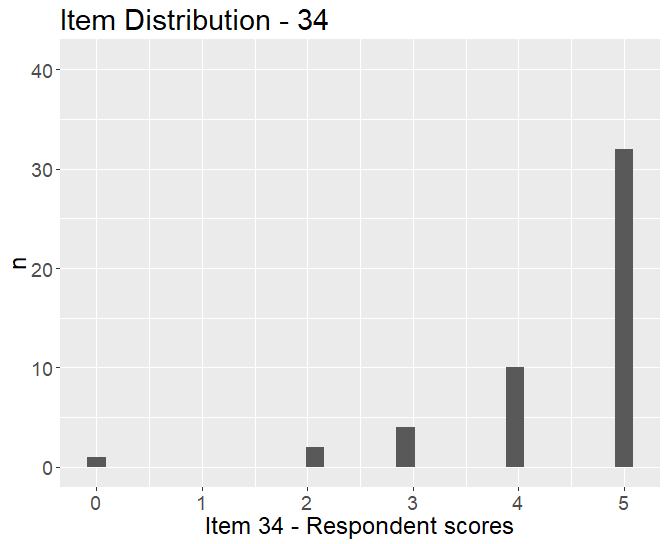 | p2_000: “Wish I could travel to visit my daughter” (Score: 3)  p2_011: “I try, I do my best to look after myself so I can go to events” (Score: 2)  p2_027: “travelling in our campervan” (Score: 5)  p2_031: “Enjoy and have a lot to do with grandkids” (Score: 5) |  |
| 35: I feel stressed – I feel calm | 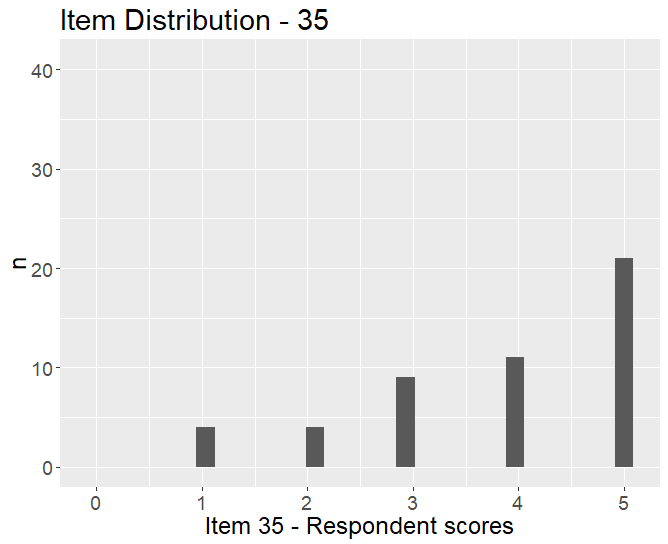 | p2_000: “I hate feeling unwell all the time.” (Score: 1)  p2_009: “Sometimes” (Score: 3)  p2_011: “stressed when I see people upset I've let them down” (Score: 2)  p2_031: “Most of the time but there is a but” (Score: 4) |  |
| 36: I find it hard to cope when bad things happen - I cope well when bad things happen | 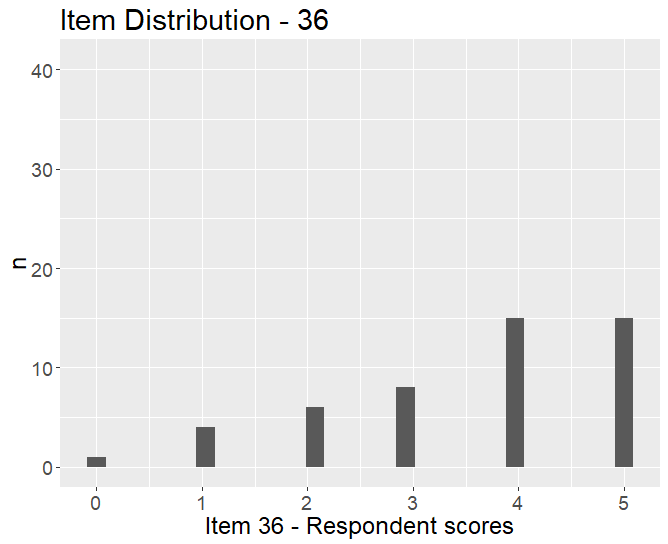 | p2_000: “Depends on the things (bad)” (Score: 3)  p2_011: “I like to help and make people feel better” (Score: 2)  p2_028: “maybe?” (Score: 5) | p2_015: “?” |
| 37: There must be more to life than living like I am now - I am living life to the fullest | 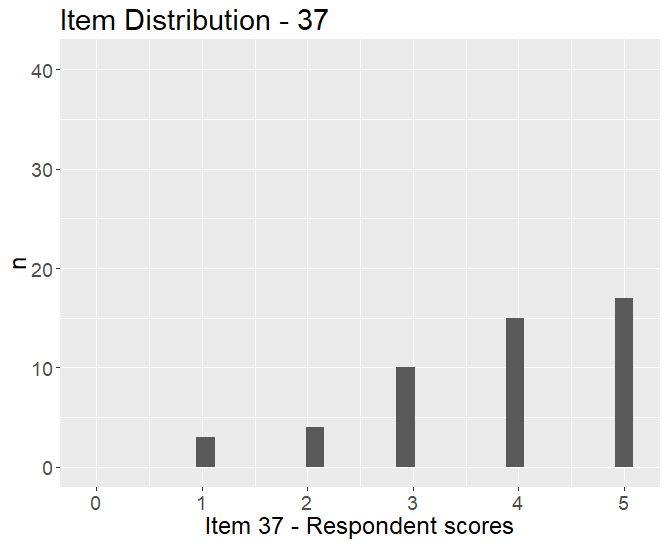 | p2_000: “True!” (Score: 3)  p2_007: “I live life to the best of my ability” (Score: 4)  p2_011: “I loved my old life” (Score: 1)  p2_031: “I have a good forward thinking feeling” (Score: 4)  p2_100: “of course but can't do anything” (Score: 3) |  |
| 38: I feel a lot older than I am - I feel a lot younger than I am | 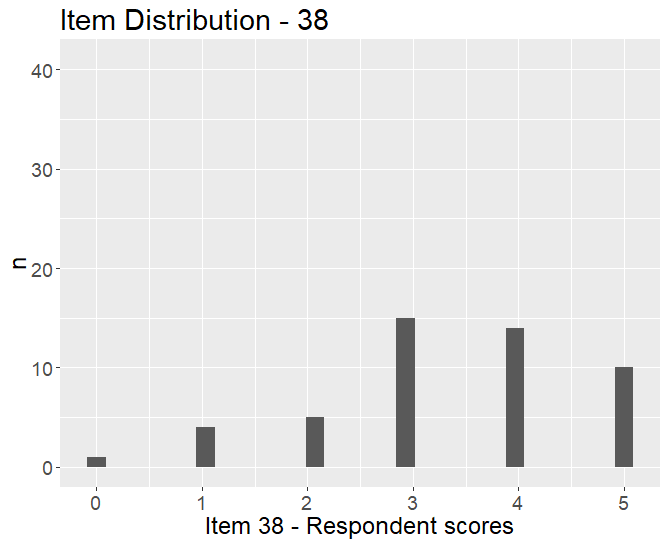 | p2_000: “Most days” (Score: 1) |  |
| 39: My health worries me - My health reassures me | 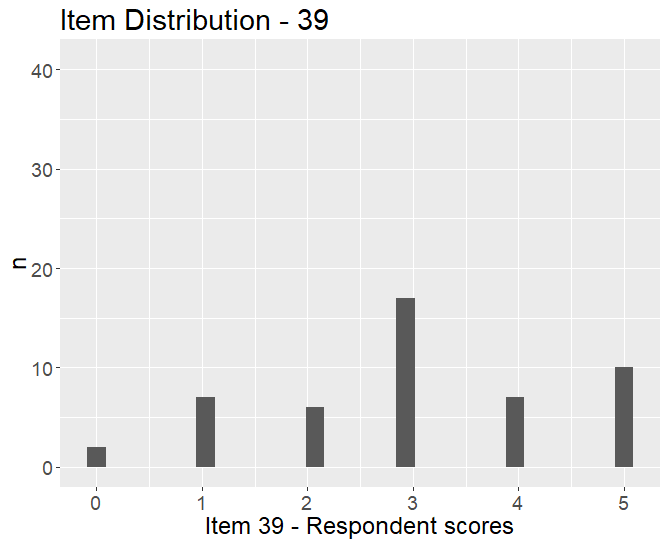 | p2_000: “I don't want my family to worry” (Score: 1)  p2_011: “I worry I'm a burden on my family” (Score: 1)  p2_031: “I do think about it maybe too much” (Score: 4) |  |
| 40: I can never forget that I have heart valve disease - It is easy to forget that I even have heart valve disease | 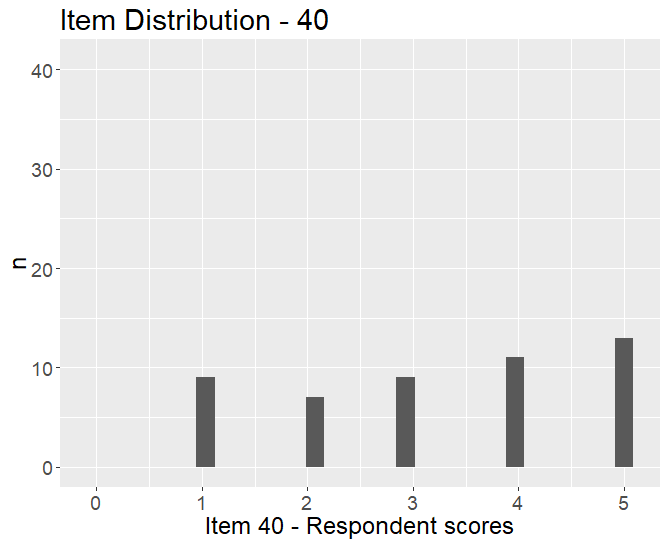 | p2_000: “Things are worse now” (Score: 1)  p2_031: “Soreness in chest reminds me. I hope it goes after 12 months.” (Score: 4) |  |
| 41: It is impossible to get the medical care I need - I can easily access the medical care that I need | 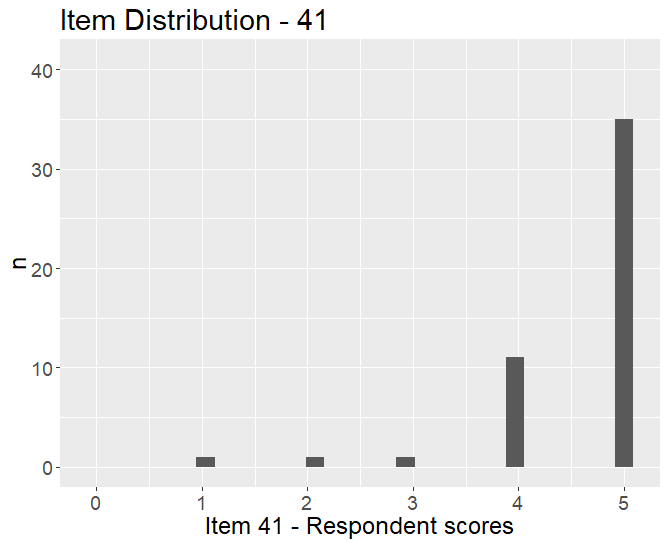 | p2_000: “Problem is to travel to Dunedin” (Score: 4) |  |
| 42: I don't trust doctors / the healthcare system - I trust doctors / the healthcare system | 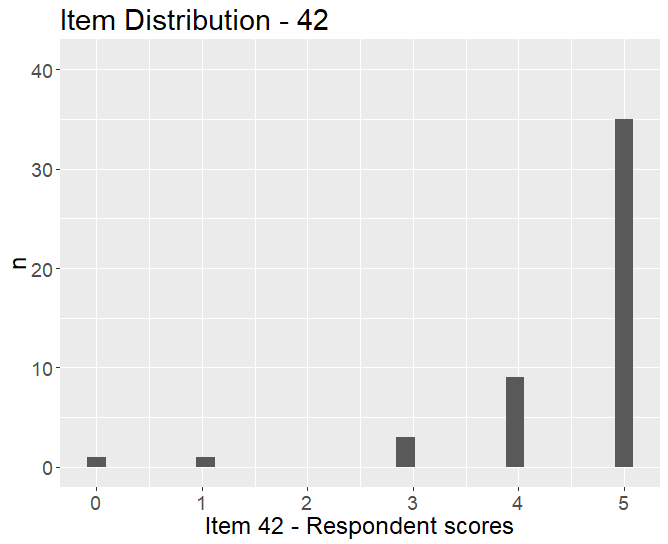 | p2_001: “[drew vertical line after 'doctors' on right hand statement] not the system” (Score: 5)  p2_011: “Amazing team, so lucky” (Score: 5)  p2_027: “Badly let down by doctors when discharged after op” (Score: 5) | “I trust my doctor but not too much the healthcare system” |
| 43: I have a negative attitude – I have a positive attitude | 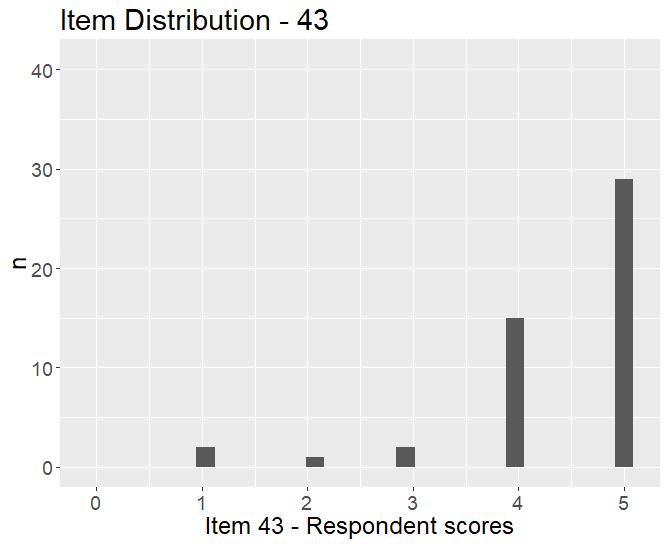 | p2_000: “It depends on the subject” (Score: 3)  p2_031: “Most of the time” (Score: 4)  p2_041: Particularly liked questions around attitude. Thought that attitude was VERY important, more so than just physical stuff. |  |
| 44: I wake up feeling tired - I wake up feeling energetic | 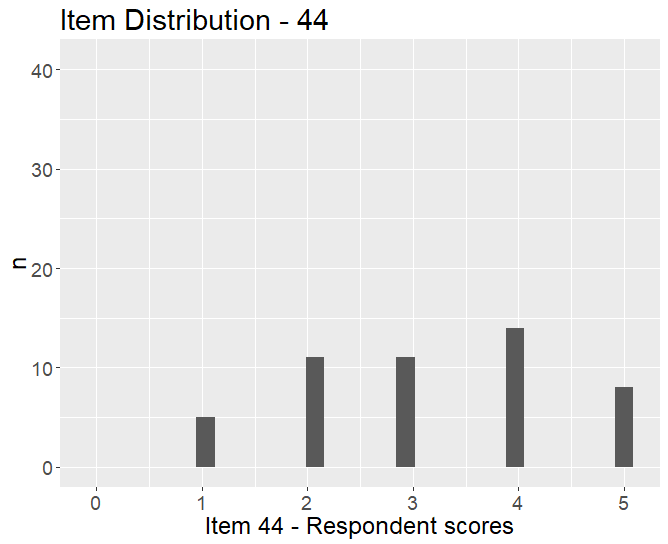 | p2_000: “Most days” (Score: 1)  p2_027: “very lucky with new valve to wake up” (Score: 3) |  |
| 45: I have no joy in my daily life - I have joy in my daily life | 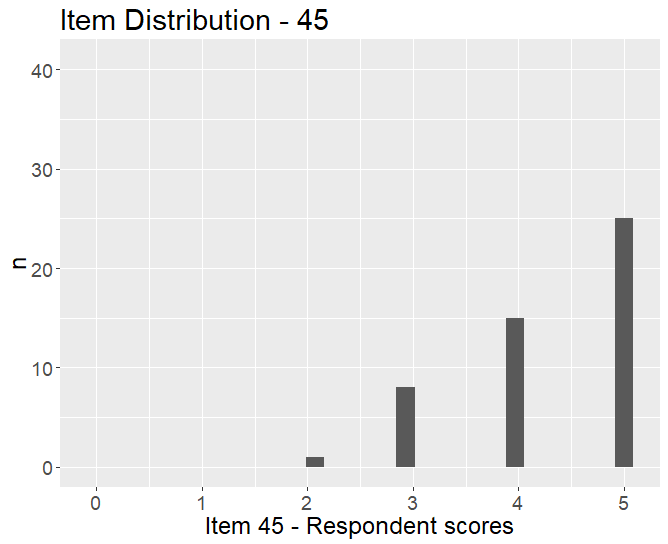 | p2_000: “It depends if I am well I am comfortable” (Score: 3)  p2_011: “My children, husband and dog. Small projects, communicating with friends” (Score: 4)  p2_031: “I like teasing” (Score: 5) |  |
| 46: I'm worried I might die away from my community - I'm not at all worried about dying away from my community | 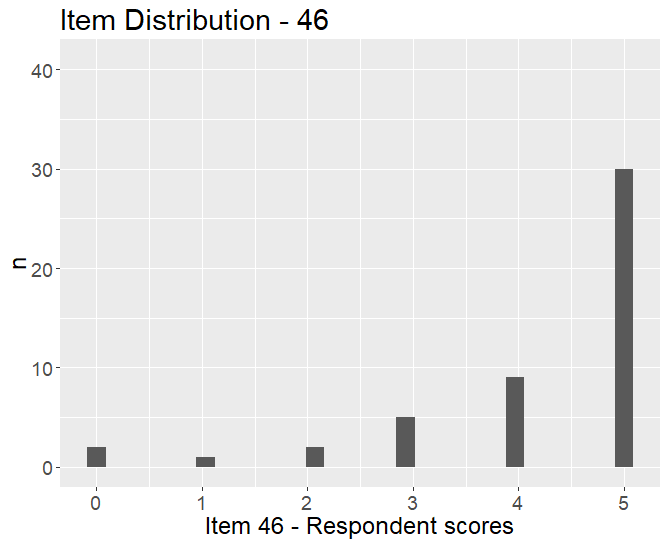 | p2_00B: “ - not my problem. Whatever will be, no choice, ha! ha!” (Score: 5) |  |
| 47: My future has been taken away from me - I have a promising future | 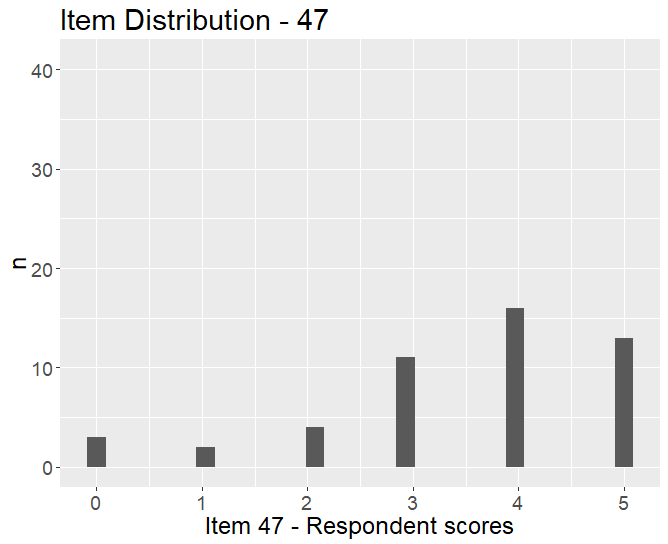 | p2_000: “I wish I was healthy and able to do things” (Score: 4)  p2_00B: “? Doing what?” (Score: 5)  p2_001: “Not because of heart disease. other health problems.” (Score: 1)  p2_011: “miss working, would like to see 70/80 years old...” (Score: 2)  p2_045: “?” (Score: 3) | p2_028: “longer live (hope)”  p2_080: “[blank] future is smaller when age gets higher” |
| 48: I'm worried about potential future operations that I might have for my heart valve disease - I don't have any worries about potential future operations for my heart valve disease | 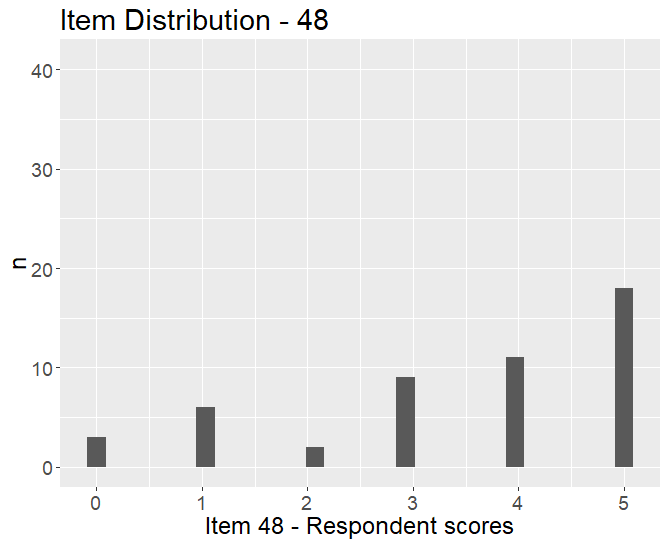 | p2_00B: “What ever will be, not in my hands!” (Score: 3)  p2_009: “As a retired nurse, I know what to expect” (Score: 5)  p2_013: “I am not accepting an operation - too old!” (Score: 1)  p2_031: “I do think about it not too much” (Score: 3)  p2_100: “Not going to have” (Score: 3) | p2_045: “told I shall not have an operation” |
| 49: My heart valve disease is a major issue - My heart valve disease is not an issue | 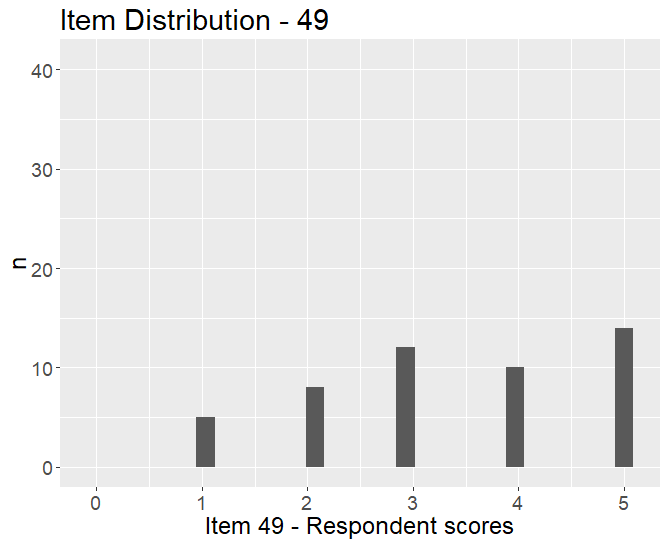 | p2_000: “Only if I have others (like blood clots)” (Score: 3)  p2_001: “The restriction of blood flow was my problem” (Score: 5)  p2_031: “I don't think about it too much. Just as well I didn't know too much about it” (Score: 3) |  |
| 50: I'm worried about the consequences I could have if I need an operation for my heart valve disease in the future (such as needing time off work, or pain) - I don't have any worries about the consequences of potential future operations for heart valve disease (such as needing time off work, or pain) | 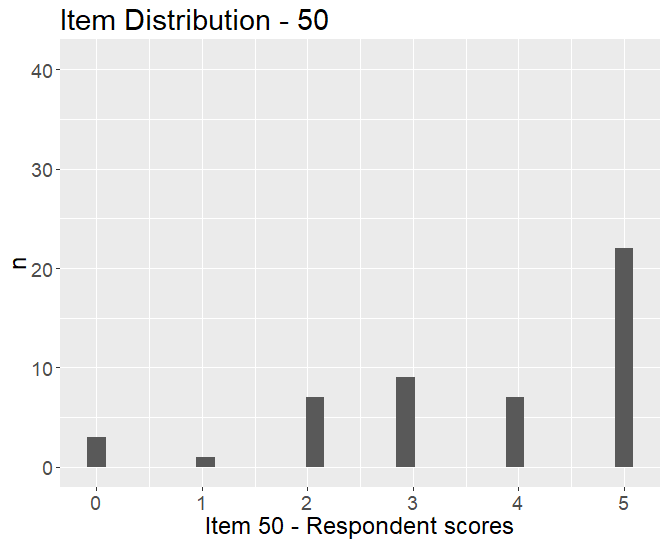 | p2_000: “I worry about recovery time” (Score: 2)  p2_00B: “OK luck may be on my side?” (Score: 5)  p2_011: “being away from children” (Score: 2)  p2_027: “retired and enjoy life” (Score: 5)  p2_031: “Keyhole surgery would be good” (Score: 4)  p2_100: “Have had an operation” (Score: 5) | p2_001: “this is the doctor call”  p2_013: “N/A”  p2_045: “not having an operation” |
| 51: There are things that my heart valve disease could do to me that are very scary - There is nothing about my heart valve disease that scares me | 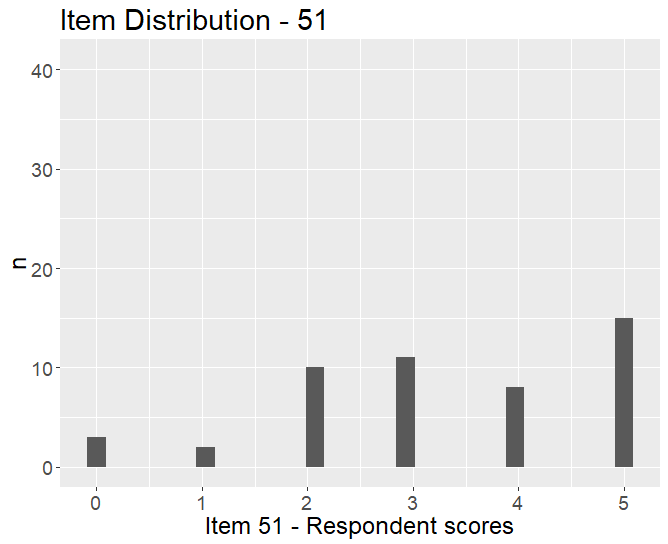 | p2_00B: “Life is scary” (Score: 3)  p2_011: “being in A-fib” (Score: 2) | p2_000: “I do not wish to linger on!”  p2_001: “same reply as q 50” [“This is the doctor’s call”] |
| 52: I am frustrated – I am content | 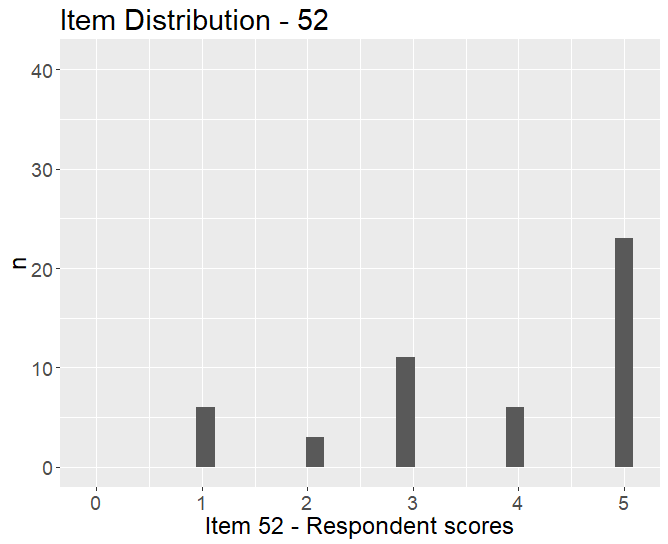 | p2_000: “Too many other health issues” (Score: 1)  p2_011: “I want to be a part of the world, working” (Score: 2)  p2_031: “Most of the time” (Score: 4) |  |
| 53: My heart valve disease has taken away who I am and made me a different person – I don’t let my heart valve disease stop or define me | 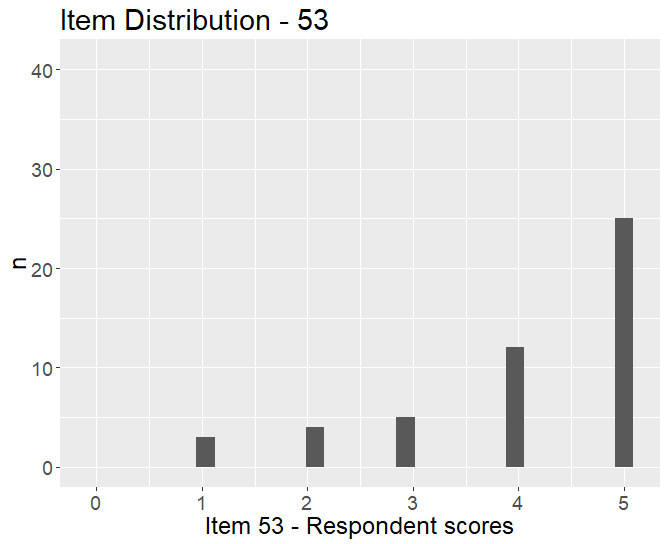 | p2_000: “I prefer not to worry about it!” (Score: 4)  p2_011: “I'm less tolerant about my children and I feel bad. Love seeing family and friends, social/community work” (Score: 2) |  |
| 54: I feel unwell – I feel well | 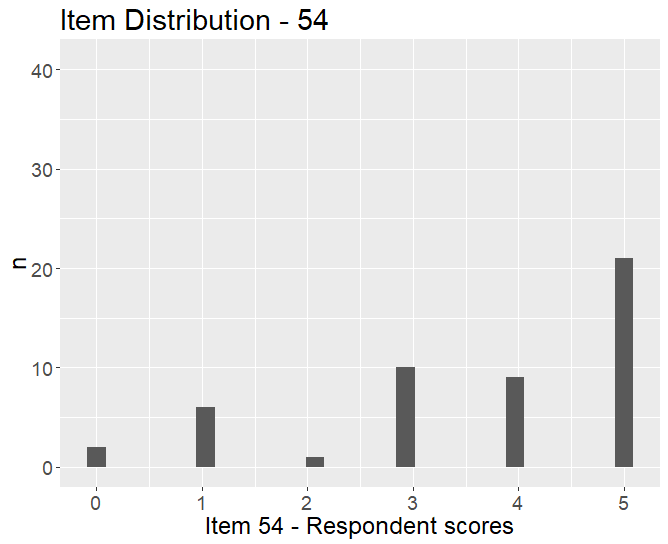 | p2_000: “More days than I wish” (Score: 1)  p2_031: “I feel chest wound could have done better” (Score: 4)  p2_032: “subject to compatible pharmaceuticals“ (Score: 4)  p2_056: “Sometimes” (Score: 3) |  |
| 55: The best treatment and management for my heart valve disease is unclear - The best treatment and management for my heart valve disease is very clear | 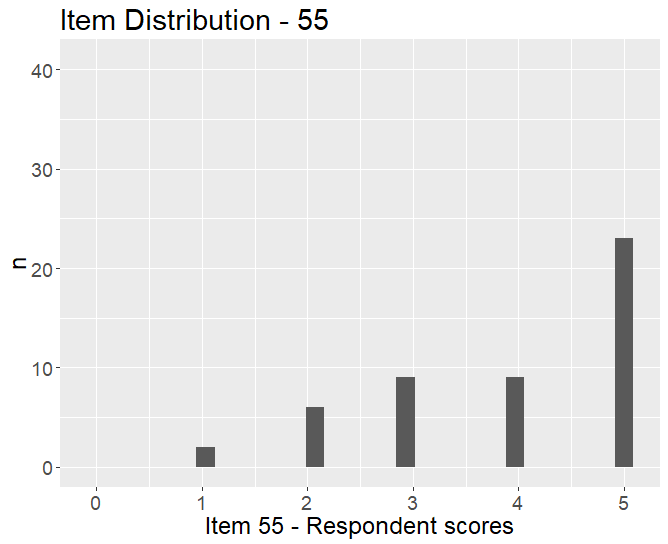 | p2_001: “I'll leave it to the doctors.” |  |
| 56: I feel aimless – I have a sense of purpose | 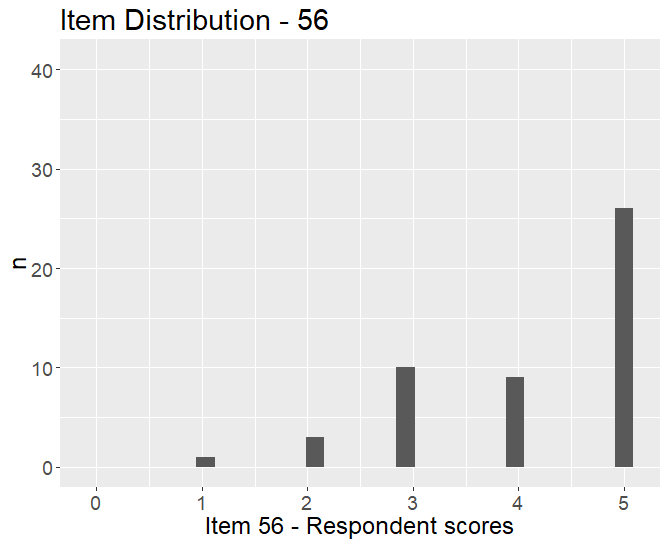 | p2_000: “Some days” (Score: 3)  p2_011: “I want to be a good mum, wife, friend, sister.” (Score: 2)  p2_041: “Aimless / no motivation” (Score: 5) |  |
| 57: I don't know what I need to do to achieve my best health - I know what I need to do to achieve my best health | 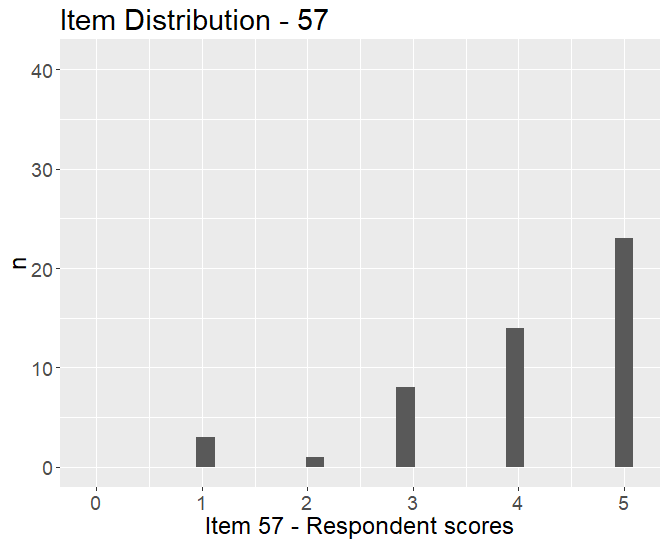 | p2_000: “I wish I have positive thoughts” (Score: 1)  p2_032: “work with GP for success” (Score: 4) |  |
| 58: I feel isolated – I feel supported | 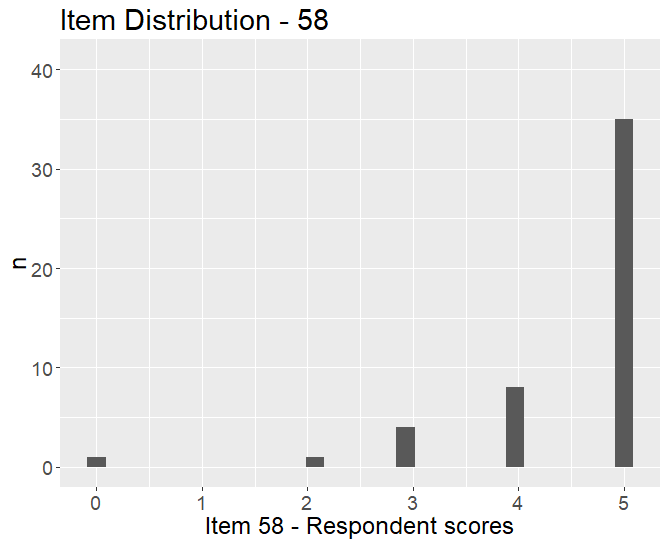 | p2_000: “Some days when I am alone” (Score: 3) | p2_009: “Sometimes |
| 59: I expect to die from my heart valve disease - My heart valve disease isn't going to seriously affect my health | 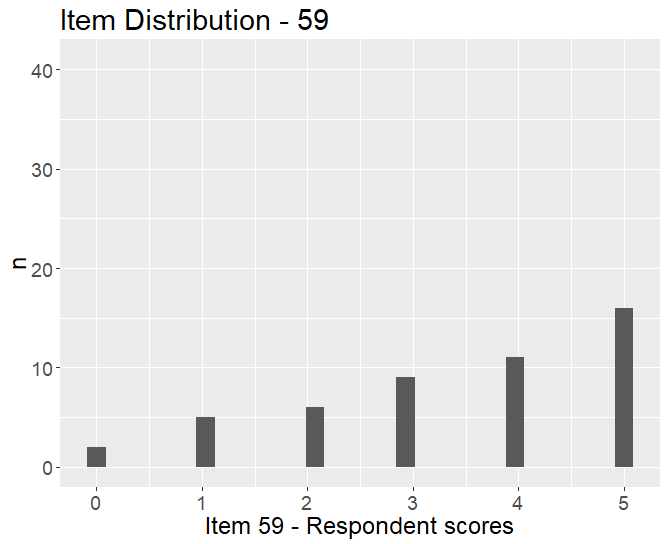 | p2_000: “I hope I do” (Score: 2)  p2_00B “?” (Score: 3)  p2_015: “I hope” (Score: 5)  p2_026: “The life span is 15 to 19 years. (I'm nearing the end of valve life expectancy: 2 years left 23rd december)” (Score: 2)  p2_027: “not now i've had replacement” (Score: 5)  p2_031: “Not totally sure about this” (Score: 4) | p2_001: “don't know doctor's call” |
| 60: I cannot control my heart valve disease and I am powerless to change my future - I am in control of my heart valve disease and my future | 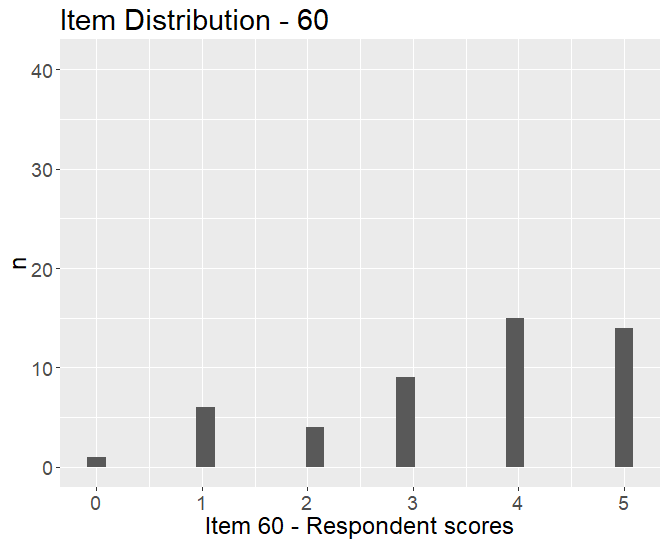 | p2_000: “I'll leave it to the doctor” (Score: 3)  p2_00B: “? Hopefully” (Score: 3)  p2_011: “I can take medication and follow doctor's advice” (Score: 2)  p2_015: “On heart medication.” (Score: 4)  p2_027: “I hope” (Score: 5)  p2_031: “I think I am” (Score: 4) |  |
| 61: Walking is difficult – walking is easy | 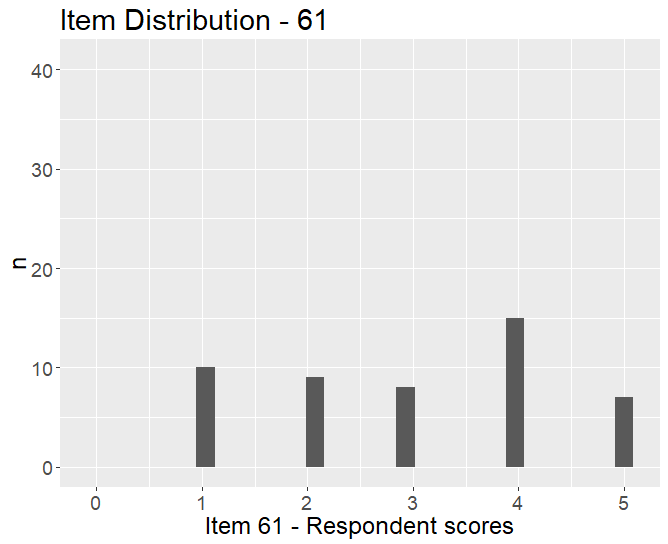 | p2_000: “Wish I could walk longer distances” (Score: 3)  p2_001: “restriction of blood from other health problems” (Score: 1)  p2_007: “Have a walking frame. Activities are restricted by my back” (Score: 3)  p2_013: “but slow” (Score: 5)  p2_025: “on the flat is fine” (Score: 2)  p2_028: “but still like biking” (Score: 5)  p2_031: “Mainly do swimming pool” (Score: 4)  p2_041: “? other problems to do with chest and heart” (Score: 41)  p2_080: “If I take it easy” (Score: 4) |  |
| 62: My pain restricts me – I am not restricted by pain | 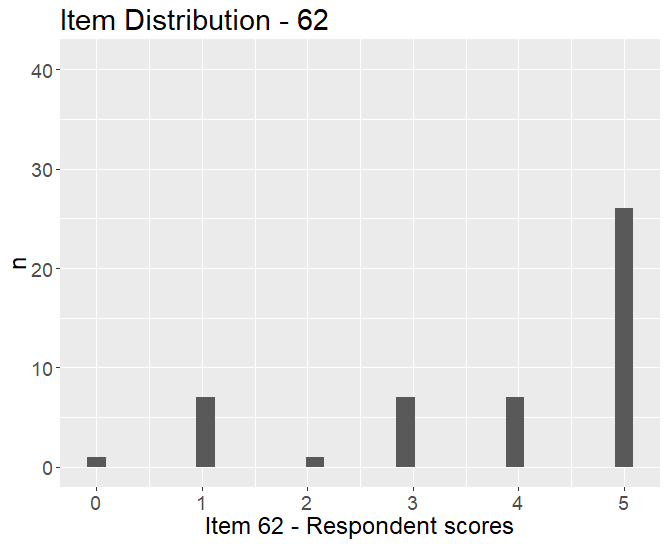 | p2_000: “I get tired quickly” (Score: 1)  p2_015: “Never had pain” (Score: 5)  p2_026: “(shortness of breath)” (Score: 3) |  |
| 63: I feel tired – I feel energetic | 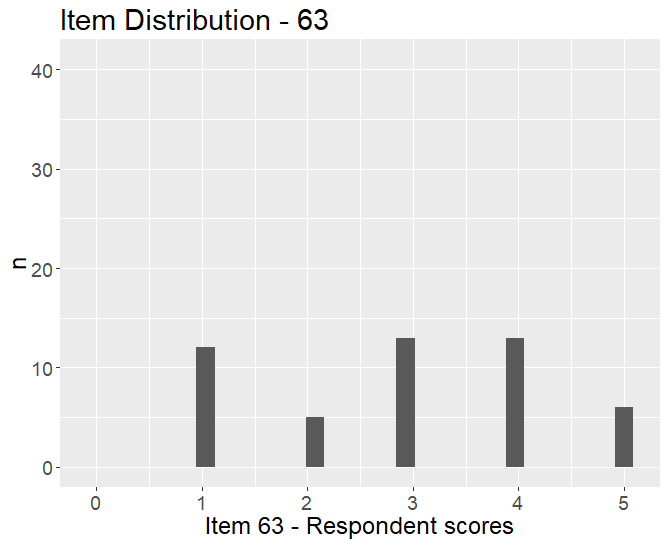 | p2_000: “Some days”  p2_013: “mostly”  p2_027: “sometimes is perhaps overdone exercise day before”  p2_031: “I do get tired in the afternoon” |  |
| 64: I have to factor in my heart valve disease every time I make decisions or plans - I can ignore my heart valve disease when I make decisions or plans | 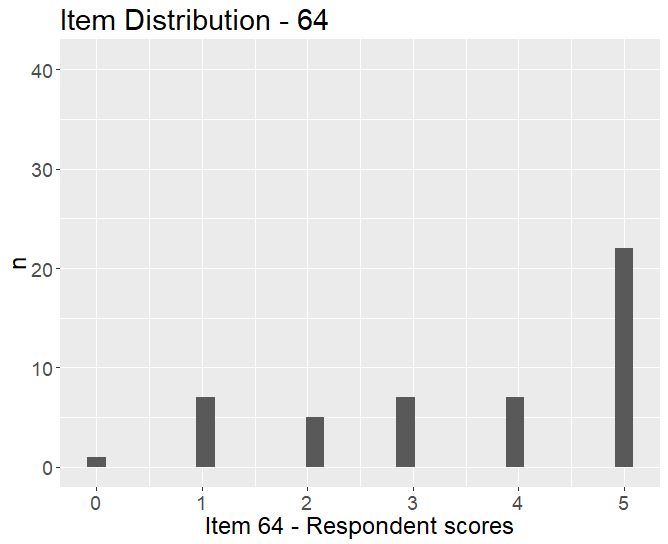 | p2_000: “I would like to travel again”  p2_031: “Most of the time”  p2_055: “No”  p2_108: “No but at almost 92 I am perhaps not your best bet” |  |

*Appendix Table Four: Original Questionnaire Items, and their Generation from Interview Findings*

| TOPIC | LEFT STATEMENT | RIGHT STATEMENT | EXEMPLARY QUOTES OR SUMMARIES OF PARTICIPANTS’ REPORTS |
| --- | --- | --- | --- |
| **Limitations** | I feel embarrassed because I need help even in basic activities | I have no need to feel embarrassed about my health | - “would hate to be dependent on someone” - “when you can’t do certain things, like walk to the bathroom….that then becomes associated with this really low quality of life” |
|  | I need help for even basic tasks | I have physical dependence in basic tasks |  |
|  | I can’t look after myself | I’m able to look after myself |  |
|  | I am unable to do my usual activities | I am able to do my usual activities | - QOL = “being able to do your usual activities" - QOL = “doing what you used to” - Physical limitations in form of being unable to manage chores and keep household up to their standards, which impacts sense of personal pride - QOL = “if someone can live comfortably the way they like to, with no problems going about their daily issues” - Career-driven and busy but now limited by HVD symptoms and unable to live as did |
|  | I’m unable to do what I used to | I’m able to do what I used to |  |
|  | I am unable to keep my home up to my standards | I’m able to keep my home up to my standards |  |
|  | I am unable to have a lifestyle that meets my standards | I am able to have a lifestyle that meets my standards |  |
|  | I’m unable to do the things I like doing | I’m able to do the things I like doing | - Less able to engage in hobbies due to HVD |
|  | I am very limited | I have no limitations | - QOL = “a life lived with no restrictions” - QOL = "being able to do things" - HVD restricts life vs not affected by HVD, asymptomatic or otherwise able to live as usual |
|  | My HVD restricts my life | My HVD does not restrict my life |  |
|  | Life is difficult | Life is easy |  |
|  | I am limited I what I can do by my health | My good health allows me do many things |  |
|  | I’m unable to do my own thing | I’m able to do my own thing | - QOL = “Doing your own thing” - HVD has cumulative effects; key result is impact on ability to live independently - Limitations of HVD directly impacting plans, or uncertainty as to whether symptoms will occur or not causing worry that causes people to cancel plans |
|  | I feel trapped by my physical limitations | I can do whatever I like |  |
|  | I’ve had to cancel a lot of my plans because of my HVD | I can do whatever I choose |  |
|  | I’m no longer able to exercise as much as I used to | I’m able to exercise more than I used to | - Symptomatic people losing exercise capacity vs. regaining it after VR - HVD often impacts ability to play sports and reduces physical capacity |
|  | I am unable to play sport | I am able to play sport |  |
|  | My challenges overwhelm me | My challenges are manageable because I take good care of myself | - Self-care makes challenges more manageable - Knows various strategies but doesn't "use them enough" - Religious faith helps preventing feeling overwhelmed |
|  | Have to change life to manage with weakness | I do not feel weak, or it does not impair me | - Continuing to work despite illness helps QOL |
|  | I expect my life and activities to be limited by my disease for a long time | I am not limited by my disease, or I expect my limitations to be over soon | - Expecting symptoms to be fixed with VR - Still not back to full health after VR - Impact of age: expecting worsening health overall even if HVD is treated |
|  | I don’t know if my limitations will last for the rest of my life | I think I will get better | - Uncertainly as to how long limitations will be present |
|  | I feel upset because my limitations have taken away things important to me | I am not limited by my disease, or my limitations have not affected what is important to me | - “having physical pain or limitations in one area, that reminds you of what you once were capable of and are no longer. I think that can evoke regret, and a bit of grief in terms of what of once was”, AS_007 - Were career-focussed and high achieving but limitations of HVD have caused loss of prior identity |
|  | My HVD as limited me so much that I’m a different person | My HVD has not affected who I am |  |
|  | Walking takes a lot more energy than it should | Walking is easy | - Walking even short distances (e.g. across room) is very difficult due to fatigue; feels like ‘walking through porridge’ or a ‘swamp’ |
|  | Walking is difficult | Walking is easy | - Able to walk long distances vs. can only walk on flat terrain - Able to walk a lot more since valve replacement |
|  | I am unable to keep up with my friends when we do things together | I am able to keep up with my friends when we do things together | - Sometimes have trouble keeping up with friends on group trips - Age is confounder; limitations may be due to age not HVD |
|  | I am able to do less than the people around me who are my age | I am able to do more than the people around me who are my age |  |
|  | My HVD makes it harder to cope when other bad things happen | I am fully able to cope when other bad things happen | - “I’m not really on top of these things enough to take it as it comes”, VR_004 - Limitations from HVD make it harder to cope with other stressors - Hope, retaining identity vs. despair - “I think a reasonable amount of stoicism would sum up my character”, VR_011 - Positive attitude, positive self-identity - Overwhelmed by limitations; unable to differentiate between limitations from HVD and personal failings |
|  | My troubles have stopped or defined me | I don’t let my troubles stop or define me |  |
|  | I can’t get through hard times | I’m able to get through hard times |  |
|  | I’m the kind of person who fails | I’m the kind of person who is a problem-solver |  |
|  | Feeling tired or unmotivated stops me from doing things | I am able to make myself to things, even when I feel tired or unmotivated | - Motivation key for QOL - Getting on with doing things even when tired/unmotivated |
|  | I’m able to do a lot less than doctors said I would | I’m able to do a lot more than doctors said I would | - Expected fewer limitations after VR but still limited after |
|  | My achievements are much smaller than they used to be | My achievements are much bigger than they used to be | - Was a busy, high-achieving person prior to HVD but now limited, has had to stop down from roles, and has difficulty achieving even small tasks of daily living |
|  | I am only able to achieve very small things nowadays | I can still achieve big things |  |
|  | I never imagined that getting older would be this hard | I'm not surprised by the limitations that result from getting older, and I'm okay with them | - Expecting poorer QOL with age - Disease causing poorer QOL, but is accepted as part of aging - Symptoms: HVD vs age? |
|  | I can't or I'm not allowed to drive a car | I can and am allowed to drive a car | - Being unable to drive is very frustrating and can cause major limitations |
| **Symptoms: SOB** | I get shortness of breath even when sitting quietly | I do not get short of breath, or it only impacts when exercising and goes away at rest | - SOB as primary symptom vs. no SOB - SOB limits activity and causes frustration/distress vs. “not bothered by it” - Worst QOL = symptoms at rest |
|  | SOB at rest | I don’t get SOB at rest |  |
|  | My SOB stops me from doing what I want to do |  | - Activity limited by symptoms; sometimes directly, sometimes due to worry inspired by potential symptom onset |
| **Symptoms: Pain** | In a lot of pain | Not in pain | - Primary and/or most limiting symptom - Limiting pain was main treatment goal |
|  | In more pain than expected | Feels prepared for pain, has resources needed to deal with it | - Recovery after VR can be painful, especially for RHD - Didn’t realise how painful VR would be |
|  | I expect my pain to get worse | I expect my pain to get better | - Living with constant level of pain; expecting it to remain |
|  | Doctors didn’t inform me of how much pain I would be in now | Doctors informed me of how much pain I would be in now | - “I definitely wasn’t fully informed about the amount of pain included [for VR]” |
|  | I get chest pain even when sitting quietly | I do not have chest pain | - Worst QOL = symptoms at rest - Constant pain vs. no pain |
|  | I'm in pain, even when I'm sitting and resting | I'm not in pain |  |
|  | I'm in pain and I don't understand why | I'm not in pain / if I am in pain, I understand why | - Not upset by pain; 'it's understandable' |
|  | Walking is painful | Walking is easy | - Severe chest pain when walking vs. no chest pain |
|  | Everything hurts. I'm tired of this | I have a positive attitude | - QOL = attitude; the way challenges are dealt with, not the challenge itself - Strong link between physical and emotional pain - Hating pain |
|  | My pain ruins the plans I make | I'm not in pain / if I am in pain, it doesn't stop me from doing anything | - Pain limits activity vs. ignoring pain and continuing on vs. no significant pain - QOL = pain-free - Emotional pain from loss of function / limitation with age - ‘Pain tolerance increases with age’ vs ‘Pain increases with age’ - Pain can be demotivating |
|  | My pain limits what I can do | I'm not in pain, or it doesn't limit me |  |
|  |  |  |  |
| **Symptoms: Fatigue** | I feel fatigued even when sitting quietly | I do not feel fatigued | - Fatigue is primary / most limiting symptom - Fatigue can be very severe - Fatigue is almost constant – becomes ‘usual’ - QOL = ‘Having the energy to do things’ - Fatigue is a nonspecific symptom and can be subtle - Had hoped to spend retirement on life outside work, but limited by fatigue |
|  | I always feel tired | I never feel tired |  |
|  | I feel fatigued at least some of the time | I never feel fatigued |  |
|  | I'm tired and it's draining. I've lost motivation | I feel energetic |  |
|  | I’m unable to keep moving and I feel weak | I’m able to keep moving and I feel energetic |  |
|  | My symptoms make it hard to do basic activities | If I do have symptoms, they don't affect what I do |  |
|  | I’m frustrated by my lack of energy | I have plenty of energy | - Limitation of basic activities drastically impairs QOL and is frustrating - Enjoys achievement, emotionally devastated by limitations of fatigue - No longer pushing self because of episode of severe fatigue |
|  | My tiredness stops me doing things that are important to me | I have enough energy to do the things that are important to me |  |
|  | I don’t have the energy to do what I need to do | I have the energy to do what I need to do |  |
|  | I don’t have the energy to live my life the way I like | I have the energy to live my life the way I like |  |
|  | I can’t get out of bed in mornings | I can get up as early as I like | - Feeling energetic when waking up vs. feeling unwell and tired when waking up |
|  | I feel exhausted, even ill, when I wake up in the morning | I wake up in the morning feeling healthy and energetic |  |
|  | I can’t even be bothered getting up and getting dressed in the morning | I wake up in the morning with lots of energy | - So fatigued that unable to do get out of bed and do basic tasks like getting dressed in the morning |
|  | I am too mentally tired to even recognise and greet friends when passing | I have the mental energy to recognise and greet friends when passing | - Recognising people easily - Having the energy and mental quickness to greet friends when passing |
|  | I feel distracted | I can focus on the task at hand | - Noting an improvement in cognitive function after treatment - Fatigue presenting as impaired concentration |
|  | I can’t concentrate for long | I can concentrate for long periods |  |
|  | Spending time with anyone, even my friends, is exhausting | I enjoy spending time with my friends | - Fatigue means spending time with anyone, even friends, is exhausting |
|  | I'm tired, and I can't fix it because I'm too tired to make plans that will help | I am able to make effective plans for when I’m fatigued | - Planning ahead helps manage fatigue, but planning ahead is difficult when fatigued |
| **Symptoms in general** | I have severe symptoms from my HVD | I don’t have any symptoms from my HVD | - Severely symptomatic vs. asymptomatic |
|  | I don’t know when my symptoms will occur | I know when my symptoms will occur | - Symptoms are unpredictable; don’t know when they will occur, and this causes negative impact on QOL |
|  | My HVD is inconsistent. I don’t know how I’ll be feeling tomorrow. | My HVD is consistent. I know how I’ll be feeling tomorrow |  |
|  | I don’t understand why I have these symptoms | I understand why I get my symptoms | - Differences in health literacy between participants; not understanding cause of symptoms can make them more distressing |
|  | My symptoms scare me | My symptoms don’t scare me | - Fear inspired by symptoms could be worse than symptoms themselves |
| **Health/Disease Perception** | I feel unwell | I feel well | - Feeling well vs. feeling ill - Many HVD patients asymptomatic - QOL likely to be bimodal: most people fine, minority very low, - Wellness is holistic and overlapping; physical, mental, emotional, and spiritual; as in Te Whare Tata Wha |
|  | I feel emotionally unwell | I feel emotionally well | - QOL = “mentally well” |
|  | I know I’m not okay | I know I’m okay | - Feeling ‘okay’ and confident vs. feeling that they’re not okay, and that life is falling apart - Have been sick for so long have forgotten what ‘wellness’ feels like - HVD causes progressive limitations - Hope is important for QOL |
|  | It’s hard to see a positive outcome for me | It’s easy to see a positive outcome for me |  |
|  | It’s hard to imagine me being well | It’s easy to imagine me being well / I am well |  |
|  | I’ve been sick for so long can't imagine what self in 'good health' feels like any more | I have been in 'good health' for a long time |  |
|  | I expect my health to get worse over time | I expect my health to get better over time | - “Not too keen on the pattern… In a few years, will I be puffed after 10 minutes?” - Expecting improvement with treatment vs. expecting decline from disease or from increasing age |
|  | My HVD cannot be fixed | My HVD can be fixed | - Viewing HVD as a purely mechanical issue and therefore as something that can be fully fixed, vs. having poor heart function before repair predicts poorer recovery |
|  | My disease makes me feel a lot older than I am | I feel a lot younger than I am | - Feeling as if they’re a lot older than they are due to their disease |
|  | I consider my heart valve disease to be a big, major issue | My HVD is just a part of life and does not affect me at all | - "[my HVD is] just a part of living", VR_003 - “don’t consider [my HVD] is major”, MR_005 |
|  | I am worried about my health | I am reassured by my health | - “I’m starting to worry about my health – especially the heart”, MR_001 |
|  | My HVD defines who I am | I don’t let my HVD define me | - Limitations from HVD so severe that they have destroyed personal identity vs. viewing self as ‘stoic’ and maintaining a positive attitude - “you have to think about [your HVD] first before you have to go and do something”, MR_008 |
|  | My HVD has stopped me | I’m determined that my HVD is not going to stop me |  |
|  | I don’t know what I need to do to achieve my best health | I know what I need to do to achieve my best health | - “I don’t know what I would need to do to get well”, MR_004 |
|  | There is no clear route for the best treatment/management for me | The treatment pathway for me is simple | - Medical system can be very confusing and communication from medical staff can be poor vs. smooth pathway for VR and well-communicated treatment plan |
|  | My progress towards health has been slow | My health is good, or improving well | - Noting improvement every day after intervention - TAVI and SVR reach same outcome at 6 months, but SVR longer recovery in the short term - Health after intervention largely depends on baseline health |
|  | I don't know enough about my disease to explain it to others when I need | I know enough about my disease to explain it to others when I need | - Being able to explain one’s disease is important when it will impact interactions with others, especially since limitations are inconsistent; feel judged by other people for inconsistent limitations |
|  | The people around me do not understand my disease and the limitations as I have as a result | The people around me understand my disease and the limitations as I have as a result |  |
|  | I expect to die from my HVD | I do not expect to die from my HVD | - Viewing HVD as something that can be easily fixed or only a minor vs. worrying about or even expecting to die from HVD |
|  | Imagining what my heart valves must look like right now is very upsetting | I am very happy with the state of my heart | - Feels distressed when imagining what their diseased heart valves looks like |
|  | I have nothing to look forward to in the future | I have things to look forward to in the future | - Need being able to “look forward to something in the future”, MR_007 |
|  | My heart valve disease has taken my future away from me | My future looks positive | - Feeling that their limitations from their HVD have taken their future away from them |
|  | I can’t see much of a future for myself, if nothing changes | My future looks positive | - “I can’t see much of a future, if nothing improves” vs having positive expectations of the future |
|  | I don’t think I can get through my HVD | I’m confident I can get through my HVD | - Overwhelmed by limitations vs. no limitations, or managing well despite limitations |
|  | Life is bad | Life is good |  |
|  | I cannot control my disease; I am powerless to change my future | I am in control of my disease and my future | - In control of their disease and their future |
|  | My HVD is just one of many problems | My HVD is my only problem / I have no problems | - Feeling overwhelmed because they have many stressors to cope with other than just their HVD |
|  | I am disappointed at my current health; I expected to be better | My current health is better than I expected it to be | - "I expected to be better quicker”, VR_004 |
|  | I’m currently worse than I was when I was at 'best health' earlier in life | I’m currently at my 'best health' | - Feeling ‘fixed’ by VR, now optimistic/secure vs. in young HVD patients, VR is not a 'fix' - Feels that they are now inherently different to ‘healthy people’ |
|  | I feel that healthy people are different to me | I am a healthy person. |  |
| **Activity** | I wish I was more active | Happy with my level of activity | - Frustrated with limitations vs. happy with / excited by level of capacity - Frustrated that current activities are less than before |
|  | I am not physically active | I am physically active | - ‘Keeping active’ is important for QOL |
|  | I sit around a lot | I keep busy |  |
|  | My daily routine is a source of stress | My daily routine helps me to cope | - Keeping up a daily routine helps them to cope with HVD vs. limitations mean that activities of daily living are a stressful challenge |
|  | Exercise makes me feel worse | Exercise makes me feel better | - Feeling better after having exercised vs. feeling excessively fatigued after exercise |
|  | I do not enjoy the physical activity I do | I enjoy the physical activity I do | - Able to enjoy activity vs. symptoms make activity a difficult challenge |
|  | My reduction in activity has ruined my life | Enjoying life even if activity reduced | - Not minding small impairments vs. frustrated - Considers limitations to be ‘just age’ and not frustrated |
|  | I'm not eating well | I am eating well | - QOL is achieved by keeping active and eating well - QOL helped by healthy, scientific practices, e.g. avoiding coffee to reduce anxiety |
|  | I don’t have any goals to work towards | I have goals to work towards | - “gotta have some goals and dreams” - Stimulating, challenging activity |
|  | Everything is boring | I have mental stimulation and challenges | - Enjoys challenging mental stimulation - Habit is comforting but novelty is fun - Novelty is a mentality that can be brought into mundane situations |
|  | My life is boring | I have novelty in my life |  |
|  | I don’t want any complex challenges; I have enough to deal with | When faced with complex problems, I’m able to enjoy the challenge |  |
| **Sleep** | I am not sleeping well | I sleep well | - Palpitations keep them awake at night and cause worry vs. not worried by palpitations - Good sleep is vital for QOL - Sleep impaired by anxiety or SOB - Activities in day are limited by tiredness - Sleeping better after their VR |
|  | I sleep worse than I used to | I sleep better than I used to |  |
|  | I can't get the sleep I need | I get plenty of sleep |  |
|  | I'm too worried or anxious to sleep well | Going to sleep is easy |  |
|  | My heart valve disease or worries about it keep me awake at night | I have no problems getting to sleep at night |  |
| **Social** | I feel like I am letting my community groups or clubs down | I feel appreciated in my community groups or clubs | - Doesn't want to be a burden - Feeling like letting sports teams down due to impaired fitness - Have had to leave professional sports team due to HVD - High-achieving; able to support others |
|  | I feel like a burden | I do not feel like a burden |  |
|  | Because of my heart valve disease, I’ve had to let people down | People can rely on me |  |
|  | I am unable to participate in volunteering or community events | I am able to regularly participate in volunteering or community events | - Able to volunteer - Able to participate in community events regularly vs. not |
|  | My work colleagues make things harder | I have good relationships with my work colleagues | - Good relationships with colleagues - Professional network valuable for maintaining QOL |
|  | I have poor relationships with people around me | I have good relationships with the people around me | - Limitations of HVD can prevent social engagement vs. feeling supported by many people |
|  | The people around me make life harder | The people around me make life easier | - Family helps when feeling stressed/down, - Not very social; happy by themselves, - Dealing with big groups of people requires energy, confidence |
|  | The people around me drain me | The people around me energise me | - Negative behaviour by other people is de-energising |
|  | I miss my friends. | I'm able to spend as much time as I want with my friends | - Missing friends - Appreciates being amongst people - Talking to others is helpful for QOL |
|  | I feel isolated and unsupported | I have people around me that I can talk to when I need |  |
|  | Had no visitors when sick or in hospital | The people around me make an effort to reach out to me when I'm sick | - Visits by family are important for QOL - Camaraderie with other patients helps QOL; ‘extra tough’ when no visitors |
| **Family** | I've had to miss important family events | I've been able to attend important family events | - Keeping tabs on family members, being interested in their careers, - Spending time with family members, being close and connected - Family is the most important for QOL, but not everyone has a connected family - Family can help QOL even when not physically around; its about the relationship - “couldn’t even watch my son’s football match” due to limitations from HVD |
|  | I feel distant from the people I love | I feel close and connected with the people I love |  |
|  | I’m not able to spend time with my family | I’m able to spend time with family |  |
|  | I am too tired or symptomatic to engage in activities with family | I can engage in busy activities with my family |  |
|  | My role in my family is to take on burdens | I feel supported by my family | - Has to take on burdens for family; finds family is a source of impaired QOL |
|  | I'm worried about dying away from home | I’m not worried about dying away from home | - Patients in rural communities worry about dying in hospitals away from home and family; unwilling to have intervention if requires leaving community |
| **Work** | I feel overwhelmed at work | I can take time for a rest at work if I need it | - Starting to having to take things slowly due to HVD/age - Having time to rest, and not rush vs. overwhelmed |
|  | I’m overworked | I’m not overworked |  |
|  | My work (or my day, if I'm retired/unemployed) is boring | My work (or my day, if I'm retired/unemployed) is creative | - Being busy / creative/ motivated - Helping others is a useful distraction from own problems, |
|  | I’m not able to be productive at work | I’m productive at work | - Being able to work vs. prevented by HVD - Has worked very hard all of life; work is part of identity - Work is more important than family - Work is a place of difficulty due to HVD - QOL = “being able to work”, AS_003 |
|  | I am unable to work (for a job, or for retirement hobbies) | I am able to work (for a job, or for retirement hobbies) |  |
| **Environment/ Circumstance** | I can't afford things I need | I can afford a comfortable lifestyle | - Being able to afford a comfortable lifestyle helps QOL |
|  | I feel like I'm stuck in a bad situation | I feel like I'm in a good situation in life | - HVD patients too high-risk for intervention have worst QOL - Feeling that VR would not alleviate anxiety - Potential need for VR in future induces anxiety - Noticeable improvement in symptoms in the first week after VR - Too high-risk for intervention: poorest QOL |
|  | I am frustrated with my situation | I am happy with my situation |  |
|  | There are a lot of stressful things going on | Things are simple and easy | - “[HVD was] only a part…Just the straw that broke the camel’s back”, MR_004 |
|  | I am unable to find solutions to problems in my life | I can find solutions to problems in my life | - Feeling overwhelmed vs. asymptomatic and no other issues - Self-perception of someone who has a positive attitude vs. despairing |
|  | Getting to hospital when I need it is a lot of trouble | Getting to hospital when I need it is easy | - Biggest impact of HVD was life disruption of hospital visits - Longer hospital stays are more stressful/disruptive than shorter - Getting to and from hospital can be difficult - RHD patients in particular benefit from treatment being available in their own community |
|  | I don't have access to the healthcare services I need | I have access to the healthcare services I need |  |
|  | It's hard for me to get treatment because it means I have to leave my community | The treatment I need is provided in my local community |  |
| **Concerns** | I have reduced confidence in doing physical activity; I fear it's unsafe | I know how much exercise is 'safe' | - Uncertain what level of physical activity is ‘safe’; decreased confidence causes activity reduction - Activity can be worried by specific fear of ‘dropping dead’ - Worry is induced as unsure if harmless symptoms are pathological and due to HVD; unable to forget diagnosis even when busy in the day vs. ignoring worries - VR tends to restore activities that had been reduced due to worries - Diagnosis and treatment were so sudden that had no time to be worried - Diagnosis causes feelings of unease, being upset, stress, worry vs. diagnosis accepted completely and calmly - General anxiety over health |
|  | Because of my diagnosis, I now worry about symptoms that wouldn't have bothered me before | I don’t worry about my symptoms / I don’t get symptoms |  |
|  | Constantly worried about HVD even when busy in day | Not worried about HVD, or only occasionally |  |
|  | My diagnosis makes me feel distressed | It was a relief to get my diagnosis |  |
|  | My diagnosis is constantly in the back of my mind | I sometimes forget I even have heart valve disease | - “[One’s diagnosis is] always at the back of your mind”, MR_008 |
|  | Because of my diagnosis, I now worry about symptoms that wouldn't have bothered me before | I’m not worried about my symptoms | - I’m never really sure whether that’s just due to my fitness or whether it’s actually a heart thing”, RHD_001 |
|  | Seriously worried about dropping dead from HVD | Not worried about dying from HVD | - AS patients are most likely to have worry of suddenly ‘dropping dead’ - “am I now gonna kark it?”,MR_002 |
|  | Seriously worried about dying from pulmonary oedema | Not worried about dying from HVD | - “Would be an awful thing to happen…Eventually your lungs fill up with fluid, and that’s it”, AS_005 |
|  | My heart valve disease could cause me to die in a nasty way, and I’m afraid of that | Nothing about my heart valve disease scares me | - Worries about dying suddenly, dying from pulmonary oedema, or dying after failing to reach achievements and living life in a dissatisfying way |
|  | I’m afraid of dying | I’m not worried about dying | - Fear of dropping dead vs hadn’t even come to mind - As increase age and friends start to die, awareness of own death increases |
|  | I can’t stop worrying about my health | My health reassures me | - “A lot of my anxiety is around my health”, MR_002 |
|  | I’m anxious about possible future surgery, treatments, or other consequences of my HVD | Nothing about any consequences of my heart valve disease scares me | - Many participants intimidated by and anxious about potential future surgeries |
|  | I’m anxious about recovery from valve replacement | I’m excited about recovery from valve replacement | - Worried about the impact recovery from VR will have on capacity and daily living - Worried about how painful recovery from VR might be |
|  | I feel anxious over unknown big events in the future | I feel excited about the future | - Anxious and uncertain about future vs future looks bright |
|  | I don't feel confident/hopeful about health today | Confident/hopeful about health today | - Worried before intervention vs. not worried about intervention - Uncertainty over future vs. positive expectations for future; looking forward to resuming activity after VR - Health worries due to age - Frustrated over slow recovery from VR vs. happy with rapid recovery after VR - Frustrated after minimal improvement from VR vs. happy with daily improvement after VR |
|  | I'm not confident/hopeful about health over the next few years | Confident/hopeful about health over the next few years |  |
|  | My life has stopped | Life goes on | - “life goes on", VR_011 |
|  | I’m afraid to leave the house because of my health issues | I feel comfortable leaving the house | - Fear of leaving the house due to increased need to urinate due to being on diuretics |
|  | I’m stuck at home | I can go wherever I like | - QOL impaired most from being “trapped at home” by HVD, MR_001 - Unable to drive |
|  | I’m worried about having an emergency from my HVD when travelling | My HVD won’t cause me any problems when travelling | - "It has crossed my mind, pretty much every trip,”, AS_007 - Increased cost of travel health insurance due to HVD |
| **Feelings** | I feel emotionally depleted | I feel emotionally nourished | - Feeling emotionally and spiritually depleted |
|  | I feel spiritually depleted | I feel emotionally depleted |  |
|  | I am full of worry | I have peace of mind | - Feeling worried vs having peace of mind |
|  | Exercise makes me feel worse | Exercise helps me feel less stressed | - “If I’m feeling a bit lethargic I probably go and do some exercise, that will always pick me up”, AS_006 - Stress management can include exercise |
|  | I am full of worry | I am mindful | - Stress management can include: exercise, mindfulness, routine, deep breaths, stoicism |
|  | Even my breathing seems wrong | I always take calm deep breaths |  |
|  | I am easily upset | I am stoic |  |
|  | I get migraines from my stress | My stress doesn’t bother me | - Migraines from stress |
|  | I feel stressed | I feel relaxed and prepared | - Feeling stressed vs relaxed - Stress negated by preparation |
|  | I feel stressed by things outside my control | I have control over my life | - QOL more impaired when have no control of stressors, and/or stress is out of control |
|  | My daily routine is stressful | My daily routine helps me feel less stressed | - Routine can help manage stress but stressors every day reduce QOL |
|  | I feel emotionally unstable | I feel emotionally stable | - QOL = “emotionally stable”, AS_007 |
|  | I feel anxious | I feel calm | - QOL = “peace within myself”, AS_007 |
|  | There is no laughter in my life | There is laughter in my life | - Having laughter in daily life is key part of good QOL |
|  | I am frustrated | I am content | - “frustration; I guess that’s the biggest thing”, RHD_001 |
|  | I feel irritable | I feel calm and patient | - Feeling calm vs irritable |
|  | I don’t have anything to cope with my anxiety / negative thoughts | I have effective strategies to cope with my anxiety / negative thoughts | - ACT therapy: observing thoughts (both pain and joy) rather than changing them |
|  | I feel grumpy | I feel satisfied | - Feel grumpy |
|  | My anxiety is out of control | I am in control of how I feel | - “There’s a level of anxiety or fear that starts to interfere with your capacity to rationalise”, AS_004 |
|  | My symptoms make life miserable | If I do have symptoms, they are easy to manage | - HVD made their life ‘miserable’ but QOL improved with VR - Limitations in what can achieve causes frustration / emotional pain |
|  | I consider myself to be a person who worries a lot | I consider myself to be a person who doesn’t let things worry me | - “I don’t go down those negative thoughts”, MR_004 |
|  | My HVD has ruined my life | Life has continued as normal | - “life goes on", VR_011 - Severe fatigue causing feeling that life is ruined and cannot achieve goals - ‘Shaken’ by HVD but made decision to carry on |
|  | My life and my goals are derailed | I am able to reach my goals |  |
|  | If things continue like they are, then my life won’t be worth living | I have a good life | - Suffering very poor QOL, with negative perspectives for the foreseeable future |
|  | There must be more to life than living like I am now | I am satisfied with my life |  |
|  | I don’t have a sense of purpose | I have a sense of purpose | - Having a sense of purpose |
|  | I feel sad | I feel happy | - Mood impacts QOL |
|  | I have to work for things I don't even care about | I put my energy into the things that matter to me | - Finding exhaustion positive because it indicates the day was productive - Being able to do things they care about |
|  | I am unsettled / things are wrong | I am content | - sense of unease and vague wrong-ness |
|  | There is no joy in my daily life | There is joy in my daily life | - QOL = “joy” in life |
|  | I feel angry at myself for wasting time | I am satisfied with the amount of work I get done | - Anger at self for low productivity |
|  | I have no motivation | I feel motivated | - Poor QOL can cause poor mood, causing poor motivation - Feeling motivated and productive promotes QOL |
|  | I feel sad. I'm hurting, emotionally. | I feel well emotionally | - QOL = "being happy in yourself”, MR_001 - Feeling of emotional hurt |
|  | If I feel unhappy, I feel that life is terrible | I am able to feel that life is good even when I’m not happy | - “I’m pretty lucky, you know. I’m happy in myself, happy by myself”, MR_001 - QOL helped by ‘remembering all things pass’ and that ‘life goes on’ - “it’s just your attitude. You’ve gotta remind yourself that every day is a good day”, VR_005 |
|  | I have a negative attitude | I have a positive attitude |  |
| **Medications/ Treatment** | I don't trust my medications | I know my medications help | - Concerns over quinine, warfarin because are poison in other applications - Patients not worried about warfarin, but doctor worried about bleeding risk |
|  | I haven’t been given the medications I need | I have been given the medications I need | - Feeling that they don’t have the medications they need to achieve good health |
|  | My medications make life harder | My medications help | - Medications promoting health vs the burden of taking them (especially anticoagulants) |
|  | The instructions of how to take my medication are complicated or annoying | My medications are simple to take | - Feeling emotionally flat due to medication vs. medication having no side effects - Some medications have severe side effects and have to be stopped - RHD treatment requires painful injections - Medications are troublesome/annoying to take, e.g. specifications on taking with meals/water and at specific times, requiring diet changes vs. medications are no trouble to take - Anticoagulation requires INR monitoring, which takes up a lot of time and requires a lot of visits |
|  | I have to spend a lot of time getting treatment/tests for my HVD | I don’t have to spend much time getting treatment/tests for my HVD |  |
|  | Treatment for my HVD can be very painful | Treatment for my HVD isn’t painful |  |
|  | My medications/treatment have caused severe/dangerous side effects | My medications/treatment don’t have any severe/dangerous side effects |  |
|  | My medications cause me a lot of trouble | My medications are no trouble |  |
|  | I don't take my medication | I take my medication as instructed |  |
| **Healthcare experience** | Doctors just tell me things without listening to what I think | Doctors ask for my opinion | - Significant distress caused by crudely-given / overly blunt diagnosis by doctor, lack of sympathy, or their symptoms being taken seriously by a doctor - VR can be intimidating and a good explanation by an engaging doctor is reassuring - Treatment for RHD is complex and has complex impacts and therefore requires engaged discussion between doctor and patient - “you feel like you’re not being understood, or they don’t know how you feel, so a lot of the time I don’t bother even mentioning it”, MR_008 |
|  | Doctors have been dismissive or arrogant with me | I’m able to discuss what matters to me with doctors | - “I know he didn’t believe that I had anything wrong with me”, MR_008 |
|  | I've been distressed by an experience I've had with a doctor | I’ve enjoyed my interactions with my doctors | - “he just had a really unfortunate manner, I mean, that’s when I got the panic attack”, MR_002 - Feeling their doctors have been very good |
|  | Doctors don’t consider how I feel emotionally | I’m able to discuss what matters to me with doctors | - Feeling that their feelings have been ignored vs having open communication |
|  | Doctors don’t have time / aren’t available for me to ask questions of them | Doctors have time / are available for me to ask questions of them | - “There’s no time in the [healthcare] system to hear all of this”, MR_004 |
|  | The language my doctor speaks makes it hard for me to understand them | My doctor speaks my first language | - Patients may have a language barrier - Doctors can be intimidating to members of minority cultures |
|  | My doctor is from a very different culture to me | My doctor looks like me |  |
|  | I’m annoyed because what doctors tell me to do is restrictive | I’m happy with what my doctors have told me to do | - Frustration with limitations imposed by medical advice |
|  | The advice/instruction I have gotten from the healthcare system makes no sense | The advice/instruction I have gotten from the healthcare system is easy to understand | - Communications from the health system can be very confusing, even conflicting |
|  | Doctors have made mistakes when treating/managing me | The doctors who treated me are good at what they do | - “was given the wrong information and diagnosis by the hospital”, FC_003 |
|  | The healthcare system is confusing | The healthcare system is easy to navigate | - Navigating the healthcare system can be very challenging, and this is only worsened with poor health literacy |
|  | I can’t access the information about my health that I need | I have access to the information about my health that I need | - Feeling in control of their health information and able to find out what they need vs feeling blocked by a hard-to-navigate health system |
|  | I have been failed by the healthcare system | The healthcare system has helped me | - Delays and misdiagnosis making people feel ‘failed’ by the healthcare system |
|  | Doctor's orders seem pointless, even damaging | Following doctor's orders makes them feel noticeably better | - VR reduced limitations vs. VR will reduce QOL in short term, especially RHD - Frustration when a doctor wants to change pills but patient feels they are working well - Medications have side effects but patients suffer worse QOL if they do not take them - Trust in the health system and long-term engagement is vital - Discovered that recovery went best when followed doctor’s orders vs. distrusts advice, gets second opinion |
|  | The instruction I have gotten from the health system has done harm to me when I followed it | The instruction I have gotten from the health system has helped me when I followed it |  |
|  | The treatment I have for my HVD is not effective | The treatment I have for my HVD is effective |  |
|  | The treatments I’ve been given are unhelpful and dangerous | The treatments I’ve been given are helpful and safe |  |
|  | I feel forgotten by doctors / the health system | Doctors / the healthcare system have taken care of me |  |
|  | I don't trust the health system | I trust the health system |  |
|  | My disease or what my doctors tell me I need to do is hard to understand | I fully understand my disease and what needs to be done about it | - Anxiety reduced by being fully informed and feeling welcome to ask questions - Having family around when being told important information is key, as they are able to remember what patient does not - Shock of diagnosis can cause patient to forget following information |
|  | I've forgotten what my doctor told me | I remember the important information my doctor told me |  |
|  | I should go to a doctor to get tests, but I haven't | I am up-to-date on medical tests and advice | - Being proactive with medical testing |
|  | I'm on a long waiting list, and it's very stressful | I can get treatment quickly | - To be finally called in for surgery after long wait was a relief - “I was on the waiting lists for five months – two more than recommended…waiting was stressful”, VR_004 |
|  | I think I was treated too late; I should have been treated earlier | I was treated at the right time |  |
|  | What my doctors told me would happen and how I would be was inaccurate | What my doctors told me would happen and how I would be was accurate | - Misdiagnosis reduces QOL and reduces trust in doctors - People often surprised by pain after VR - Complications slowing recovery after VR |
|  | I’ve had to spend a long time in hospital | I don’t have to spend much time in hospital | - Long stays in hospital are very disruptive to life |
